# Genomic Organization and Generation of Genetic Variability in the RHS (Retrotransposon Hot Spot) Protein Multigene Family in *Trypanosoma cruzi*

**DOI:** 10.3390/genes11091085

**Published:** 2020-09-17

**Authors:** Werica P. Bernardo, Renata T. Souza, André G. Costa-Martins, Eden R. Ferreira, Renato A. Mortara, Marta M. G. Teixeira, José Luis Ramirez, José F. Da Silveira

**Affiliations:** 1 Department of Microbiology, Immunology and Parasitology, Escola Paulista de Medicina, Universidade Federal de São Paulo, São Paulo 04023-062, SP, Brazil; wericabernardo@gmail.com (W.P.B.); renataepm@hotmail.com (R.T.S.); edendearaujo@gmail.com (E.R.F.); ramortara@unifesp.br (R.A.M.); 2 Department of Parasitology, Instituto de Ciências Biomédicas, Universidade de São Paulo, São Paulo 05508-000, SP, Brazil; andreguilherme@usp.br (A.G.C.-M.); mmgteix@icb.usp.br (M.M.G.T.); 3 Fundación Instituto de Estudios Avanzados (IDEA), Universidad Central de Venezuela, Caracas 1080, Venezuela

**Keywords:** *Trypanosoma cruzi*, Retrotransposon Hot Spot (RHS) multigene family, chromosome distribution, recombination, gene mosaic structure, evolution, nuclear protein

## Abstract

Retrotransposon Hot Spot (RHS) is the most abundant gene family in *Trypanosoma cruzi,* with unknown function in this parasite. The aim of this work was to shed light on the organization and expression of RHS in *T. cruzi.* The diversity of the RHS protein family in *T. cruzi* was demonstrated by phylogenetic and recombination analyses. Transcribed sequences carrying the RHS domain were classified into ten distinct groups of monophyletic origin. We identified numerous recombination events among the RHS and traced the origins of the donors and target sequences. The transcribed RHS genes have a mosaic structure that may contain fragments of different RHS inserted in the target sequence. About 30% of RHS sequences are located in the subtelomere, a region very susceptible to recombination. The evolution of the RHS family has been marked by many events, including gene duplication by unequal mitotic crossing-over, homologous, as well as ectopic recombination, and gene conversion. The expression of RHS was analyzed by immunofluorescence and immunoblotting using anti-RHS antibodies. RHS proteins are evenly distributed in the nuclear region of *T. cruzi* replicative forms (amastigote and epimastigote), suggesting that they could be involved in the control of the chromatin structure and gene expression, as has been proposed for *T. brucei*.

## 1. Introduction

The flagellate protozoan *Trypanosoma cruzi* is the etiologic agent of Chagas disease or American trypanosomiasis, which affects 6–7 million people mainly in Latin America, with an increasing number of cases in non-endemic countries such as Canada, the United States of America, and some European countries [1]. When compared with other members of the genus *Trypanosoma*, the *T. cruzi* genome was expanded, being 2.3-fold larger than that of *T. brucei* and *T. rangeli*. Repetitive DNA sequences comprise about 52% of the *T. cruzi* genome [2,3,4]. The dramatic expansion and diversification of repetitive sequences, particularly of multigene family encoding proteins, such as surface proteins (TS (Trans-Sialidase), MASP (Mucin-Associated Surface Protein), mucins, gp63, Retrotransposon Hot Spot (RHS), and DGF-1 (Dispersed Gene Family-1)) may have contributed to the speciation of the *T. cruzi* taxon [2,5]. RHS proteins are coded by a multigene family found in the genus Trypanosoma. RHS refers to a hot spot for retrotransposon insertion within the RHS gene. When retrotransposons are inserted in this site, they generate RHS pseudogenes carrying one or more retroelements flanked by two separate halves of RHS [6]. Multiple RHS genes have been annotated in the genomes of mammalian trypanosomes (African trypanosomes—*T. brucei*, *T. congolense*, and *T. vivax*; American trypanosomes—*T. cruzi*, *T. cruzi marinkellei*, and *T. rangeli*; and cosmopolitan trypanosomes—*T. theileri*, *T. evansi*, *T. conorhini*) and *T. grayi* isolated from reptiles.

RHS proteins were first identified in *T. brucei* and were classified into six subfamilies (RHS1 to RHS6) based on the C-terminal region sequence [6]. The RHS proteins of *T. brucei* share a highly conserved amino-terminal (N-terminal) region, while the carboxy-terminal (C-terminal) portion is highly variable [6]. The N-terminal region has an ATP/GTP binding motif encoded by five codons located upstream of the hot spot insertion site for the retrotransposons Ingi (an autonomous long interspersed element—LINE) and RIME (a non-autonomous short interspersed element—SINE). The pseudogene may be the result of homologous recombination between two RHS variants by crossing-over involving the 5′ region upstream of the retroelement insertion site. Retrotransposon insertion generates nonsense mutations or frameshifts within the coding sequence, resulting in truncated RHS proteins [6].

The role of the RHS family has been investigated in *T. brucei*, and it has been suggested that RHSs are involved in the control of the expansion of the retroelements in this organism [6,7]. Earlier studies in *T. brucei* showed an increase in the level of RHS transcripts after the ablation of argonaute protein, suggesting that the RHS family may be under the control of siRNA (small interfering RNA) [8]. High throughput analysis of small non-coding RNAs showed that a large number of pseudogene-derived siRNAs originated from pseudogene–pseudogene pairs, in which the major components were RHS pseudogenes [9], and it has been hypothesized that RHS pseudogenes in *T. brucei* are a source of antisense siRNAs, which regulate the expression of the RHS family. More recent studies proposed that the RHS family could be involved in the chromatin modeling [10], transcription elongation, and mRNA export in *T. brucei* [11].

Beyond an initial genomic analysis showing multiple RHS (gene) pseudogenes, little is known about the organization, structure, and expression of these genes and their products in *T. cruzi.* In the current study, we aimed to investigate the structure, evolution, and expression of the RHS multigene family in *T. cruzi*. We also provide insights into the strategies used by *T. cruzi* for preserving complete and functional RHS genes.

## 2. Material and Methods

### 2.1. Parasites

Trypanosome isolates used in this study were the *T*. *cruzi* clone CL Brener (CLB) (TRYCC426, [12], and G strain [13]), *T*. *cruzi marinkellei* (TCC344)*, T. rangeli* SC58 [14] and *T*. *brucei rhodesiense* YTAT 1.1. The epimastigotes of *T*. *cruzi*, *T. cruzi marinkellei*, and *T. rangeli* were grown in axenic cultures at 28 °C in liver-infusion tryptose (LIT) medium [15] supplemented with 10–20% heat-inactivated fetal calf serum. Procyclic forms of *T*. *brucei rhodesiense* YTAT 1.1 were cultured in a semi-defined medium (SDM-79) supplemented with 10% heat-inactivated fetal bovine serum at 27 °C. *T. cruzi* extracellular amastigotes were obtained by culture tissue trypomastigote differentiation in a LIT medium, as previously described [16].

### 2.2. Identification of RHS Sequences in T. cruzi and T. cruzi marinkellei Genome Databases

The search for homologous RHS genes in the TriTrypDB and GenBank databases was performed using the algorithms BLASTp, tBLASTn, BLASTx, and the presence of RHS domain architecture was confirmed using rpsBLAST [17]. RHS transcripts of CLB were used as queries to identify homologous sequences in other *Trypanosoma* species using the tBLASTn (e-value of 1 × 10^−3^) search program. The retrieved sequences were evaluated for the presence of RHS domains with the rpsBLAST algorithm (e-value of 1 × 10^−5^) against the database of conserved domains [18]. An extra round of tBLASTn was performed using found RHS sequences as a query to improve genome survey sensibility. Figure S1 shows the flowchart of this analysis. Sequence alignments were carried out with RHS of clone CLB excluding truncated sequences. The nucleotide and amino acid sequences were aligned using the MUSCLE program [19] and the poorly conserved regions were removed using the Gblocks program [20].

### 2.3. Classification and Phylogenetic Analyses of RHS

For these analyses, we selected RHS transcripts of the *T. cruzi* clone CLB [21]. Transcribed genes were analyzed for the presence of RHS domains with the rpsBLAST algorithm using 1 × 10^−5^ e-value against the NCBI Conserved Domain Database (CDD) [18] (Figure S1). Sequences that showed false-positive RHS domains and pseudogenes were excluded. In the phylogenetic analysis, the global multiple alignment was carried out with the MUSCLE algorithm [19]. Phylogenetic trees were generated using the “Maximum likelihood method” using the RaxML v 8.2.9 program [22], with an automatic search for substitution models (PROTGAMMAAUTO) selected by the Akaike information criterion (AIC) (auto-prot = AIC) information criterion, with 1000 bootstrap replicas. The phylogenetic tree was visualized with the program FigTree V 1.4.2 [23].

### 2.4. Detection of Potential Recombination Events in RHS Sequences

The RHS sequences selected for the phylogenetic study were also used to identify recombination events in the clone CLB using the RDP4 program (Recombination Detection Program) [24], which allows the identification and statistical analysis of recombination events from a set of aligned sequences. It uses non-parametric recombination detection methods (algorithms RDP, GENECONV, MaxChi, Chimera, Bootscan, 3Seq, and SiSscan) to identify breakpoints in the genomic sequences where recombination begins and ends, in addition to the donor parental sequences of the recombinant fragment. For recombination events, sequences detected by at least 6 of the 7 algorithms in the RDP4 package were considered recombinant.

### 2.5. Expression and Purification of Recombinant RHS

An 877-bp fragment encoding a 292-aa region of the carboxy-terminal domain of the RHS (TcCLB.511055.20) was amplified by PCR from CLB genomic DNA, cloned into pGEM-T, and sequenced to confirm gene identity. Then, it was subcloned into pGEX-1λT to produce the RHS-GST fusion protein as described by Martins et al., 2015 [24]. *E. coli* BL21 bacteria were transformed with the RHS-GST construct, grown in LB medium, and protein expression was induced with 1 mM isopropyl β-D-1-thiogalactopyranoside (IPTG). The RHS recombinant protein was extracted from the insoluble fraction of bacterial lysates with Laemmli’s sample buffer and separated on 10% SDS-PAGE. The band W to the recombinant protein was excised from the gel and extracted by dialysis against ammonium bicarbonate and distilled water [24]. The purity of recombinant RHS was checked by SDS-PAGE stained with colloidal Coomassie Blue and immunoblotting (Figure S2). Purified protein was quantified with Coomassie Plus (Pierce, Thermo Fisher Scientific, Waltham, MA, USA) in 96-well plates at 620 nm.

### 2.6. Antibody Production, Western Blot, and Immunofluorescence Analyses

About two mg of the purified RHS recombinant protein were sent to Rheabiotech Research and Development Laboratory, SP, Brazil, for the production of polyclonal anti-RHS antibodies in mice. The specificity and reactivity of the anti-RHS antibodies were determined by ELISA and Western blot assays using the recombinant protein RHS.

Epimastigotes (10^8^ cells) of *T. cruzi* (clone CLB, strain G), *T. cruzi marinkellei*, and *T. rangeli*, and procyclic forms (10^7^ cells) of *T. brucei* were washed in PBS and lysed with 4 × Laemmli’s sample buffer, and the extracts were subjected to SDS-PAGE (10% for separation gel and 3% for packaging gel) at 120 V for 45 min. Proteins were transferred to Hybond ECL membranes (Amersham, GE Healthcare Life Sciences, Foster, CA, USA). For the Western blot reaction, the membrane was blocked in 1× PBS solution containing 7.5% skimmed milk powder (PBS/milk solution) for 1 h at room temperature. The membrane was then incubated with PBS/milk solution anti-RHS1 (dilution 1:500) for 1 h, at room temperature. Subsequently, the membrane was washed three times (3 × 5 min) in PBS containing 0.05% Tween 20 (PBS/Tween solution). Secondary antibodies (Sigma Aldrich, St. Louis, MO, USA) were incubated for 1 h at room temperature at a dilution of 1:10,000. Bound antibody signals were amplified with ECL (Enhanced Chemiluminescence) substrate (GE Healthcare, Buckinghamshire, UK) and luminescent bands visualized in an Alliance 2.7 photo documenter (UVItec, Cambridge, UK).

For indirect immunofluorescence assay, *T. cruzi* epimastigotes (10^7^ cells) were harvested from the culture medium, washed with PBS, and fixed with 2% paraformaldehyde in PBS for 15 min at room temperature. Then, the parasites were washed with PBS and incubated with anti-RHS antibodies (1:1000 dilution) in the presence of 0.1% saponin and 1% PBS/BSA for 1 h at room temperature. The parasites were washed once more with PBS and incubated for 1 h with an Alexa Flour 568 anti-mouse IgG antibody raised in goat diluted 1:100 in 1% PBS/BSA and 1 mM DAPI (4′,6 -diamino-2-phenylindole, Molecular Probes). Subsequently, epimastigotes were washed with PBS and the slides were mounted using Glycerol-PPD (p-Phenylenediamine). Images were acquired with a TCS SP5 II TandemScanner confocal microscope (Leica Microsystems, Wetzlar, Germany) using a 63 × NA 1.40 PlanApo oil immersion objective and processed with Imaris software 7.0 (Bitplane).

## 3. Results

### 3.1. Mapping of RHS Sequences on the Chromosomes of Clone CLB of T. cruzi

Natural populations of *T. cruzi* reproduce predominantly by binary fission, therefore they exhibit a clonal population structure [25,26,27,28]. However, the occurrence of hybridization has been demonstrated in vitro [29] and also in natural populations of *T. cruzi* [28,30,31,32,33,34,35,36]. Based on several genetic markers, *T. cruzi* isolates were classified into six discrete typing units (DTU) named lineages TcI to TcVI [37,38,39]. The isolates from lineages V and VI have a hybrid evolutionary origin from at least two hybridization events between lineages TcII and TcIII [28,33,34,39].

The clone CL Brener (CLB) is a hybrid strain grouped in lineage TcVI, and sequence analysis of its genome revealed the presence of two haplotypes [2], one of which has contigs similar to the Esmeraldo strain of lineage TcII. The sequence divergence between the two haplotypes is 5.4% [2]. The genomic sequences generated in the Genome Project of *T. cruzi* clone CLB have been organized in 41 pairs of homologous chromosomes (TcChr), with the smallest having 77,958 bp (TcChr1) and the largest 2,371,736 bp (TcChr41) [2,40,41]. Due to the hybrid nature of CLB, each pair of homologous chromosomes consists of one homolog, which is an Esmeraldo-like-haplotype (S), and another homolog, which is a non-Esmeraldo-like haplotype (P), totaling 82 in silico chromosomes (TcChr) [2,40]. A search for RHS sequences in the CLB genome deposited in the TriTrypDB database resulted in 525 RHS sequences (111 genes, 384 pseudogenes, 30 truncated sequences), which are distributed in the haplotypes as follows: 48 complete genes, 177 pseudogenes, and 8 truncated sequences in the Esmeraldo haplotype (S), and 63 complete genes, 207 pseudogenes and 22 truncated sequences in the non-Esmeraldo haplotype (P) (Table S1). Besides these sequences, we found 42 complete RHS genes, 175 pseudogenes, and 11 truncated sequences among the unallocated contigs, totaling 753 RHS sequences in the CLB genome. RHS gene sizes range from 351 to 3014 bp. The estimated RHS content of the CLB genome was 3,271,841 bp, comprising about 5.4% of the *T. cruzi* genome sequence.

The distribution of RHS sequences along the CLB chromosomes is shown in Figure S3. Among 82 chromosomes, three chromosomes, TcChr1-S, TcChr4-S, and TcChr34-S, did not show RHS sequences. Larger chromosomes, such as TcChr40 and TcChr41, have predominantly RHS pseudogenes (Table S1), suggesting that RHS and other repetitive sequences could be involved in the expansion of the chromosome size. It is important to highlight that the total number of RHS sequences present in the genome of the CLB may be even greater than that obtained in this analysis. When non-transcribed sequences were included in our analysis, the total number of RHS sequences was larger than one thousand, showing the presence of fragments dispersed in the genome, which are reminiscent of RHS genes. These results reflect the complexity of the *T. cruzi* genome and RHS family [2,6,42]. The haploid genome of *T. cruzi* is about 2- and 5-fold larger than that of *T. brucei* and *Leishmania* spp., respectively. In addition, multigenic families (trans-sialidases, mucins, DGF-1, MASP, RHS, and GP63 proteases) underwent a very pronounced expansion process in *T. cruzi* [2,3,6,42,43,44].

**Figure 1:**
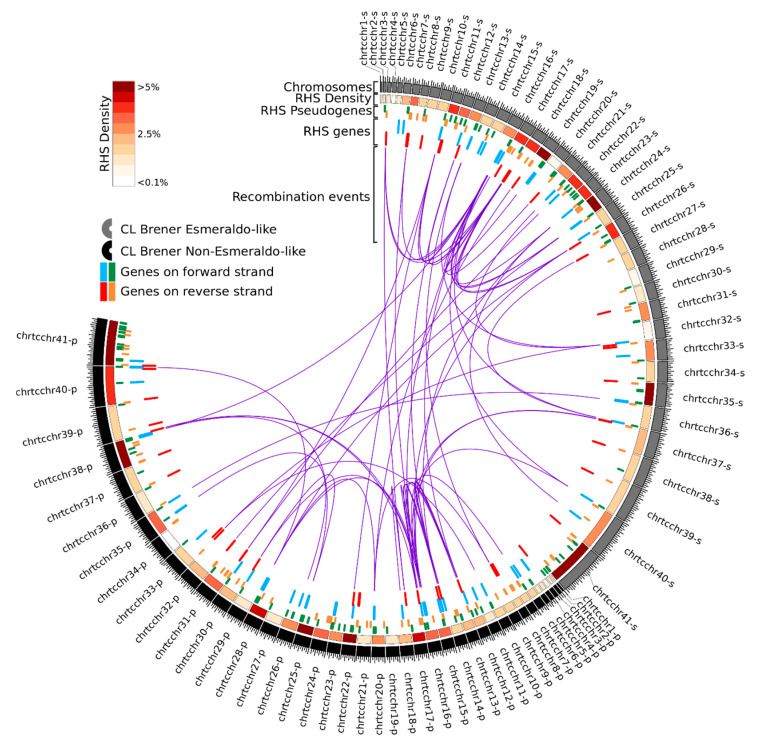
Circos diagram depicting the genomic organization and recombination events of the RHS family in the whole genome of *Trypanosoma cruzi* clone CLB. Inner track 1 represents the recombination between RHS genes. The recombinant sequences are linked to putative major and minor parental, using purple and green lines, respectively. Track 2 shows the genomic organization of RHS genes in chromosomes. Genes on forward and reverse strands are colored in blue and red, respectively. Track 3 shows the genomic organization of RHS pseudogenes in chromosomes. Pseudogenes on forward and reverse strands are colored in green and orange, respectively. Track 4 depicts a heat map of RHS genes’ and pseudogenes’ density for each chromosome. Values were obtained by summing the length (bp) of RHS genes and pseudogenes and were divided by the chromosome size. Outer track 5 shows the representation of *T. cruzi* CLB chromosomes for Esmeraldo (haplotype S) and non-Esmeraldo (haplotype P) allelic loci.

The frequency of RHS sequences in each chromosome of CLB was plotted as a heatmap in Figure 1, and the proportion of total RHS length in each chromosome is shown in Figure S4. RHS sequences comprise 0.34% to 6.14% of the entire length of each CLB chromosome. Overall, the frequency of RHS was similar in most pairs of homologous chromosomes. However, in some homologous pairs, this proportion was quite different, e.g., between the haplotypes S and P of the chromosome TcChr20 or TcChr21.

### 3.2. Phylogeny and Classification of the RHS Multigene Family of Clone CLB

**Figure 2:**
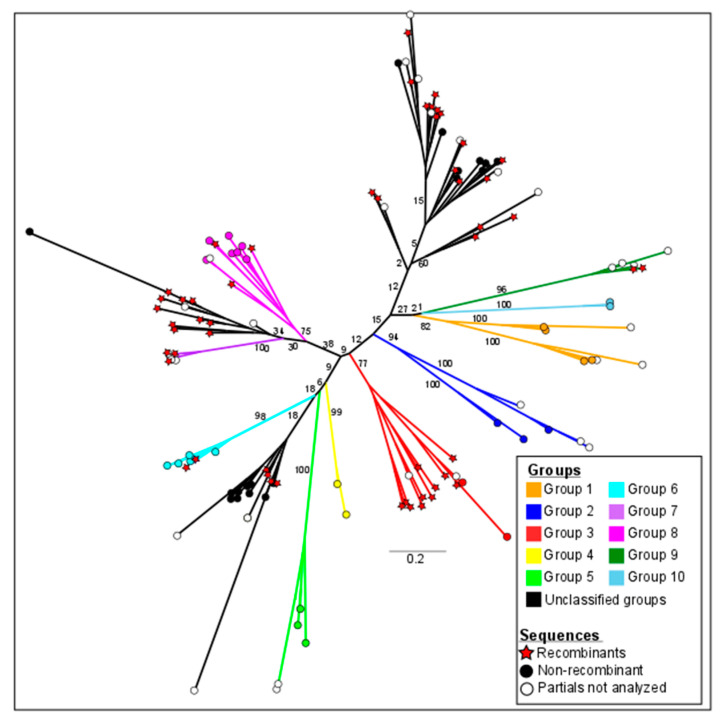
Phylogeny and classification of transcribed RHS sequences. Phylogenetic analysis was carried out using the RaxML v 8.2.9 program with an automatic search for substitution models (PROTGAMMAAUTO) selected using the Akaike information criterion (AIC) (auto-prot = AIC), with 1000 bootstrap replicates. Groups 1–10 comprise RHS sequences, with supported values separated by colors, and RHS sequences with bootstrap values below the cutoff (unclassified groups) are indicated in black.

In the phylogenetic analysis, the transcribed RHS genes were examined for the presence of RHS domains by rpsBLAST using an e-value of 1 × 10^−5^ against the database of conserved domains [18]. Aiming to reveal the real extension of recombination events within RHS genes, in this analysis, we excluded non-LTR retrotransposons or other protein families with which RHS are commonly associated. The presence of conserved RHS domains (pfam07999, PTZ00209, and TIGRO1631) was also confirmed in other databases (CDD, Pfam, SMART, KOG, COG, PRK, and TIGR). The analysis of 139 RHS amino acid sequences was carried out using the maximum likelihood method in the RxML v 8.2.9 program by replacement models (PROTGAMMAAUTO). One thousand bootstrap replicas were processed to confirm the degree of reliability of the groups, assuming bootstrap values >75. Seventy-four RHS sequences can be categorized into groups 1 to 10 with values above the cutoff (indicated in colors), while three groups comprising 65 sequences with bootstrap values below the cutoff (indicated in black) were designated as unclassified groups. The number of sequences per group ranged from two RHS sequences in group 10 (light blue) to 15 sequences in group 3 (red) (Figure 2 and Table 1). Phylogenetic analysis showed that each RHS group consists of a monophylogenetic group. The results were also shown in the format rooted in the midpoint (Figure S5), where all the sequences with their respective TriTrypDB access numbers can be appreciated [41].

The bulk of detailed information of the RHS groups of the CLB genome, such as chromosome mapping, genomic location including the subtelomeric region, the sizes of the coding sequence, and the predicted translated protein, is shown in Table 1. Most of RHS transcribed genes (70%) encode proteins of approximately 60 to 180 kDa, and the remainder encode peptides of 38 to 10 kDa. The RHS sequences selected for phylogenetic analysis were those assigned to CLB chromosomes (TcChr). Out of 74 RHS sequences, 58 genes have only one copy located in haplotype S or P, resulting in a hemizygous condition. Twenty-two of the hemizygotes are located in the subtelomere, a polymorphic region susceptible to homologous recombination, including ectopic recombination [5,45,46].

Our results showed that RHS hemizygotes can also be found in the interstitial chromosome regions in which the synteny is interrupted by a set of RHS sequences [47,48]. It has been proposed that the *T. cruzi* genome is organized in two compartments: a core compartment comprising conserved and hypothetical conserved genes, and a non-syntenic region (disruptive compartment) enriched by repetitive sequences such as members of multigene families TS, MASP, and mucins [3]. Other multigene families (GP63, DGF-1, and RHS) are dispersed throughout both compartments [3].

The subtelomeres of *T. cruzi* could be included in the disruptive compartment since they are enriched by genes encoding surface proteins (TS, MASP and DGF-1), retrotransposon hot spot genes (RHS), retrotransposon elements, satellite DNA, RNA-helicase and N-acetyltransferase genes [45,48,49,50,51]. Twenty-five chromosomal ends of CLB chromosomes (TcChr) are composed mostly of RHS genes and pseudogenes [45]. The disruptive compartment including the subtelomeric regions could act as sites for homologous recombination [2,3,5,26,28,29,30,32,33,34,35].

**Figure 3:**
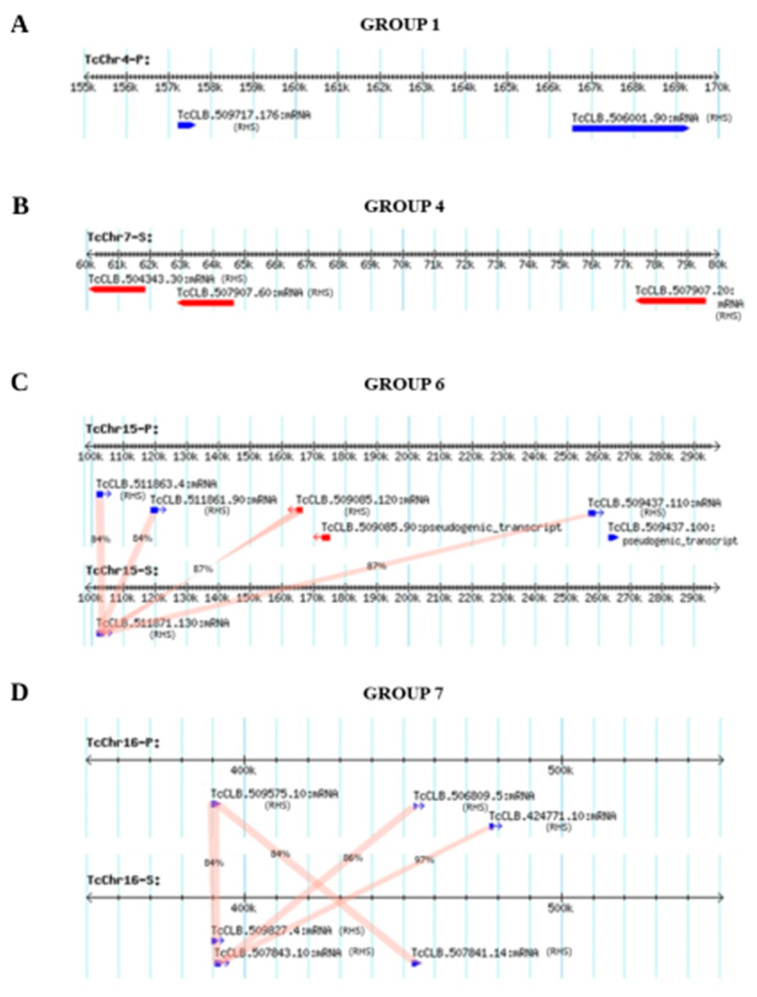
Gene duplication events in the RHS sequences of clone CLB. The figure shows the physical map of the chromosome regions involved in the recombination event. For clarity, only RHS sequences are shown. The direction of transcription is indicated by blue (sense) and red (anti-sense) arrows. (**A**,**B**) Groups 1 and 4: duplication of RHS genes by unequal crossing-over with loss of tandem counterparts in one of the haplotypes (TcCh4-S and TcChr7-P). (**C**) Group 6: duplication of the RHS genes by unequal crossing-over with the conservation of one of the RHS counterparts in the TcChr15-S haplotype. (**D**) Group 7: duplication followed by genetic conversion between paralogous genes located in the TcChr16-P and TcChr16-S haplotypes (interlocus nonallelic gene conversion). The identity between homologous RHS proteins of the P and S haplotypes is indicated in the figure. The identity between paralogous RHS proteins ranged from 93 to 100%. The physical maps showing the position of RHS sequences were downloaded from the public genome database TriTrypDB [41].

The members of the RHS groups are organized in multiple clusters at various genomic locations on different chromosomes, including the core and disruptive compartments and subtelomeres. (Table 1 and Figure 1). The distance between two contiguous RHS genes ranged from 2 to 50,000 bp and the identity from 55 to 98%, suggesting the occurrence of gene duplication by homologous mitotic recombination, as has been described in fungi [52,53]. Some rearrangements could be explained by unequal crossing-over between homologous chromatids (interhomolog crossover) leading to the loss of the tandem counterparts in one of the haplotypes. For example, the RHS genes of groups 1 and 7 located on chromosomes TcChr4-P and TcChr7-S, respectively, were mapped in only one haplotype, indicating the loss of these genes in the corresponding haplotype (Figure 3A,B).

The RHS genes of group 6 were mapped to the chromosomes TcChr15-P and TcChr15-S, and only the first gene (TcCLB.511871.130) of the cluster was present on the TcChr15-S haplotype, the remainder was lost by unequal crossing-over-recombination between homologous chromatids (Figure 3C). The homologous RHS genes of the TcChr15-P encode proteins with >93% identity with each other, and they share 84% identity with the paralogous RHS (TcCLB.511871.130) of the TcChr15-S haplotype. These results showed that duplications gave rise to RHS sequences in tandem that maintained the structure of the functional gene.

The RHS genes of group 7 located on the chromosomes TcChr16-P and ThChr16-S share 84–97% identity (Figure 3D), and this arrangement could be explained by genetic duplication followed by genetic conversion between non-alleles (interlocus nonallelic gene conversion), e.g., between the RHS genes TcCLB.507843.10 (TcChr16-S) and TcCLB.506809.5

### 3.3. Generation of Genetic Variability by Recombination between T. cruzi RHS Sequences

**Figure 4:**
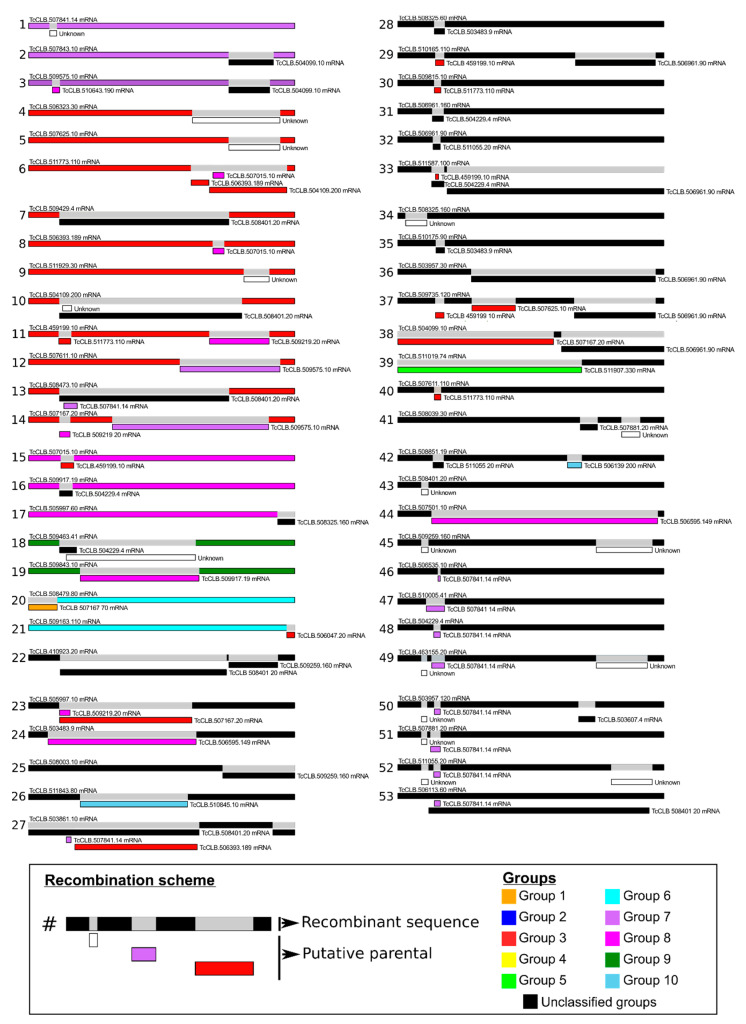
Detection of potential recombination events in *T. cruzi* RHS sequences. Recombination analysis was performed using the RDP4 program composed of non-parametric recombination detection methods by the algorithms: RDP, GENECONV, MaxChi, Chimera, Bootscan, SiSscan, and 3Seq. RHS sequences of groups 1–10 (parental sequences) are highlighted in different colors and unclassified groups (recombinant sequences) are presented in black. All RHS sequences are also indicated by their access number in the TriTrypDB [41].

In the phylogenetic analysis, we found sixty-five RHS sequences distributed in branches with low bootstrap values, which were included in the unclassified groups. Due to the high number of unclassified sequences, we investigated whether recombination events had also occurred in these sequences. We used the Circos plot to map the recombination events between RHS with a single link connecting each pair of paralogs (Figure 1). We identified 53 recombination events in 139 RHS sequences that were confirmed by at least six of the seven algorithms of the RDP4 package (Figure 4). We found that about 60% of the recombination events occurred in the unclassified sequences. Thirty-two unclassified RHS sequences were involved in the recombination events. The size of the fragment inserted into the target sequence by recombination is quite variable, and it may represent approximately 4% of the entire RHS gene. The recombination between the RHS genes results in mosaic structures that can contain up to three fragments of different RHSs inserted in the target sequence.

The recombination events occurred in different regions of RHS including the coding regions of the amino- and carboxy-terminal portions, as well as in the central region of the protein. Most recombination events were detected in the RHS sequences of group 3 that served as donors into unclassified sequences and eventually into sequences from other RHS groups. The recombination events occurred in specific regions, e.g., the amino-terminal coding region of RHS genes. As an example, the insertion of the same RHS sequence TcCLB.507841.14 of group 7 into the amino-terminal coding region of unclassified RHS sequences is shown (Figure 4, see recombination events 46 to 53).

### 3.4. Expression and Subcellular Localization of RHS in T. cruzi

The expression of RHS in *T. cruzi* and other trypanosomes was analyzed by Western blot using anti-RHS antibodies raised against a recombinant protein carrying a 292-amino acid region from the carboxy-terminal domain of RHS (TcCLB.511055.20) of CLB. This region is conserved among RHS of some *T. cruzi* strains (Dm28c, Sylvio X10/1, Y, Bug2148, Tulahuen, TCC) and *T. cruzi marinkellei*. The location of RHS (TcCLB.511055.20) in the nucleus has been experimentally demonstrated in the nuclear subproteome of clone CLB [54].

**Figure 5:**
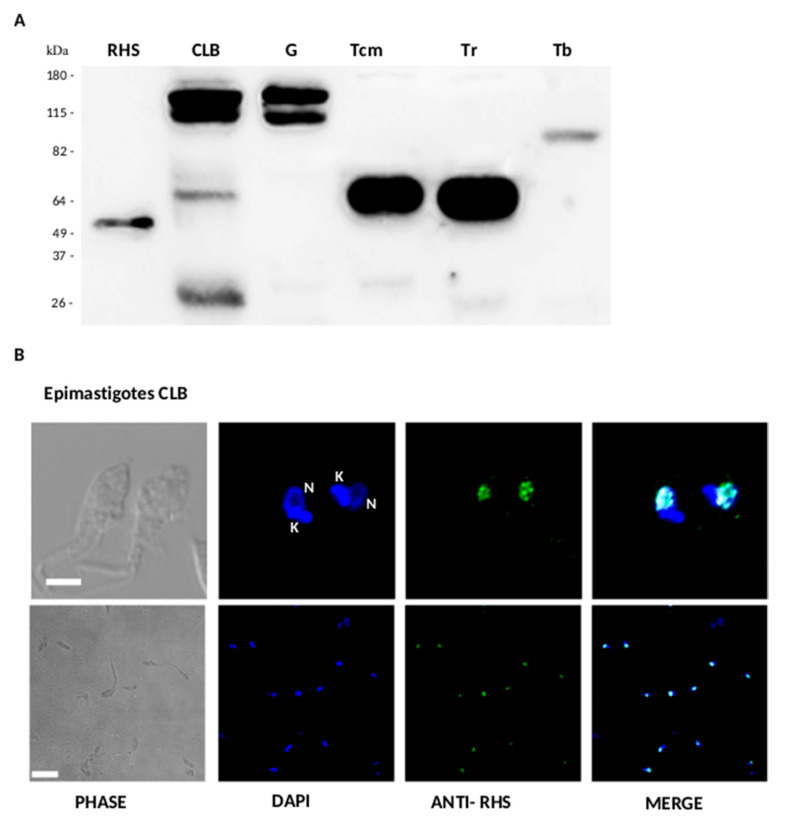
Analysis of the expression of RHS by Western blot in *T. cruzi* and other trypanosomes and cellular localization in *T. cruzi* by indirect immunofluorescence. (**A**) Protein extracts of epimastigotes of *T. cruzi* (CLB and strain G), *T. cruzi marinkellei* (Tcm), and *T. rangeli* (Tr) and procyclic forms of *T. brucei* (Tb) were separated by SDS-PAGE, transferred to nitrocellulose membranes, and incubated with anti-RHS polyclonal antibodies (diluted 1:500). The RHS recombinant protein was included as a positive control. The molecular masses of the reference proteins are indicated on the left in kDa. (**B**) Confocal microscopy images from indirect immunofluorescence reaction with anti-RHS antibodies (diluted 1:1000) in permeabilized epimastigotes of clone CLB. The labeling of the nucleus and kinetoplast DNA (DAPI) and RHS proteins is shown in blue and green, respectively. At the top, the reaction with two epimastigotes is shown at 3 μm scale. In the lower panel, the image shows epimastigotes (scale bar 10 μm). N, nucleus; K, kinetoplast.

The anti-RHS polyclonal antibodies identified different protein profiles among *T. cruzi* strains and trypanosome species. They reacted strongly with two bands of 118 kDa and 112 kDa in the *T. cruzi* clone CLB and G strain, and weakly with two additional bands of 65 kDa and 29 kDa in CLB. A single band of 65 kDa was detected in *T. cruzi marinkellei* and *T. rangeli*, and a band of 82 kDa in *T. brucei* (Figure 5A). The sizes of RHS proteins identified by Western blot are consistent with those predicted RHS ORFs in the *T. cruzi* strains and *T. cruzi marinkellei*. These results suggest that the RHS genes encoding the 118 kDa and 112 kDa proteins are expressed in the CLB and G strain, whereas the lower molecular weight (65 kDa and 29 kDa) RHS proteins are expressed only in lower amounts in CLB. *T. cruzi marinkellei* and *T. rangeli* showed a similar expression profile consisting of a single 65 kDa band. The presence of an 82 kDa RHS in *T. brucei* is in agreement with the RHS protein profile (85 to 110 kDa) described in this trypanosome [6].

**Figure 6:**
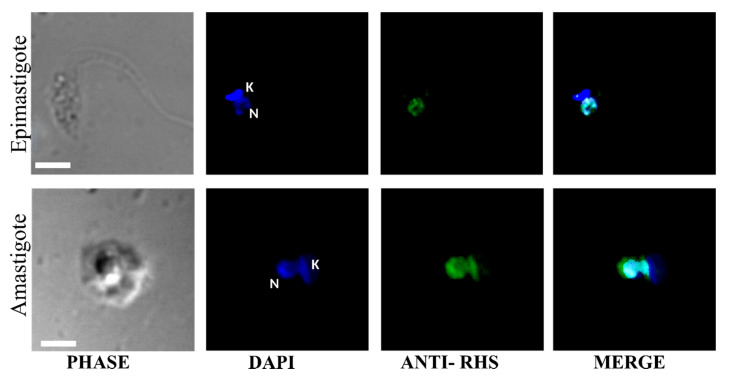
Cellular localization of RHS in the amastigote of *T. cruzi*. Confocal microscopy images from indirect immunofluorescence reaction with anti-RHS antibodies (diluted 1:1000) in permeabilized epimastigotes and amastigotes of clone CLB. The labeling of the nucleus and kinetoplast DNA (DAPI) and RHS proteins is shown in blue and green, respectively. Scale bar 3 μm. N, nucleus; K, kinetoplast.

Permeabilized parasites were analyzed by indirect immunofluorescence, using anti-RHS antibodies (Figure 5B). Nuclear and kinetoplast DNA was labeled with DAPI, and the RHS proteins were detected with fluorescent anti-RHS antibodies (shown in blue and green, respectively). The fluorescence distribution in the permeabilized parasites is concentrated at the nuclear region, confirmed by its colocalization with DAPI (Figure 5B merge). RHS distribution was concentrated in spots within the nucleus. Anti-RHS also reacted within the nucleus of intracellular amastigote (Figure 6), but no reaction was found in trypomastigotes. Taken together, these results suggest that RHS proteins of clone CLB have a predominantly nuclear location.

## 4. Discussion

### 4.1. Genomic Organization and Generation of Genetic Variability in the RHS Multigene Family in T. cruzi

RHS is a genus-specific multigene family identified in the genome of all trypanosomes sequenced so far. RHS genes have a retrotransposon insertion site in their 5′ coding region, which is predicted to disrupt more than 50% of the members of this family. Therefore, our phylogenetic analysis was restricted to transcribed RHS sequences with an uninterrupted ORF encoding the RHS domain. RHS proteins of clone CLB were categorized into 10 groups with significant bootstrap (Figure 2), suggesting that each RHS subfamily is a monophyletic group, as previously reported in *T. brucei* [6]. Regarding the unclassified RHS sequences, they were separated from the rest of the groups, suggesting some structural differentiation among these sequences, and they evolved together with other RHS groups. Our search showed that *T. cruzi* RHS paralogous genes shared 75–100% identity at the amino acid level, whereas they shared 30–47% identity with orthologous genes from other trypanosome species, such as *T. rangeli*, *T. grayi*, *T. evansi*, *T. vivax*, *T. brucei*, *T. theileri* and *T. conorhini*. From these results, we may infer that RHS genes evolved from a common ancestor and started diverging by speciation.

Once we defined the RHS sequence groups of *T. cruzi* CLB, the next question was whether recombination events occurred among the members of the various RHS groups including the unclassified ones. The comparison of transcribed RHS sequences showed the occurrence of one to three recombinational events resulting in a mosaic structure, which contains up to three fragments derived from different RHSs. The RHS sequences of unclassified groups comprised ~47% of total transcribed RHS, being involved in ~60% of the recombinational events in which they were used as a template to generate new RHS sequences. Our results suggest that the RHS family has been subjected to rapid gene turnover, resulting in different paralogous groups that are conserved for functional reasons. We believe that the unclassified RHSs may act as sequence reservoirs that can recombine with functional paralogs to generate diversity, and at the same time preserve intact copies in the RHS gene family. The lack of ancestral sequences could be explained by a continuous process of gene turnover mediated by gene conversion (allelic or ectopic) and unequal crossing-over.

The complexity of the RHS family may also be related to the large number of pseudogenes that comprise more than 50% of the family [2,6,7,42]. In *T. cruzi* and *T. brucei*, the repertoire of pseudogenes is of great importance in the generation of variants of multigenic families involved in parasitic virulence [6,55,56,57,58,59]. Taken together, these results suggest that trypanosomes developed alternative mechanisms for achieving genetic diversity in the multigene families, one of which uses incomplete genes (pseudogenes) in the generation of functional genes, while others promote recombination between functional genes. These mechanisms acting together may lead to the generation of multiple RHS sequences, resulting in the diversity within this family but preserving intact RHS copies in the genome.

Sequence diversity in the RHS multigene family of *T. cruzi* may be generated by unequal crossing-over (sister chromatid exchange and interhomolog crossover), segmental gene conversion, and interlocus nonallelic gene conversion. Tandem duplication generated by unequal crossing-over over between non-sister homologous chromatids (interhomolog crossover) may occur with the loss of tandem allelic counterparts in one of the haplotypes, leading to a condition called hemizygosity. Out of 139 transcribed RHS genes of CLB, 58 genes (~42%) have only one allele with no counterpart in the other haplotype (S or P), resulting in a hemizygous condition. We identified 22 RHS hemizygotes mapped in the subtelomere, which is a polymorphic region that is susceptible to homologous and ectopic recombination [5,45,46,49,51]. Callejas et al., 2006 [60] identified a large hemizygous subtelomere region in the chromosome I of *T. brucei*. This region accounted for three-quarters of the length of chromosome I and resulted in the amplification and divergence of gene families such as VSG (Variant Surface Glycoprotein) [60].

There is some evidence in the genome of *T. cruzi* that segmental gene conversion is involved in the generation of sequence diversity for multigene families organized in tandem array repeats [61,62,63,64]. In addition to segmental genetic conversion, we also found evidence of interlocus nonallelic gene conversion (IGC) among gene duplicates between loci. Gene conversion has been proposed as an active force in the evolution of trypanosomes [65]. Araujo et al., 2020 [66] showed that DNA replication origins in *T. cruzi* are preferentially located at the subtelomeric region, which is a site of conflict between transcription and replication that may lead to DNA double-strand breaks and generation of diversity. Wier et al., 2016 [67] suggested that gene conversion is the mechanism used by *T. brucei gambiensis* to avoid the Meselson effect of accumulation of mutations on the chromosomes for lack of sexual recombination in this species. The proposed mechanism is based on the repair of a defective gene copy on a chromosome by copying and pasting the functional gene from the homologous chromosome.

### 4.2. The Role of RHS Proteins in T. cruzi

We found that RHS proteins are located in the nucleus of epimastigotes and amastigotes of *T. cruzi*. This is in agreement with previous work [54] that identified the presence of 74 RHS proteins with apparent molecular masses of 12 to 111 kDa in the nuclear proteome of *T. cruzi* epimastigotes [54]. These data were corroborated by Western blot analysis, in which we identified RHS proteins from 29 to 118 kDa in CLB. Despite the large number of RHSs expressed in *T. cruzi*, the profile of proteins recognized by anti-RHS antibodies is relatively simple, composed of 2–3 strongly reactive proteins. A similar profile was described in *T. brucei*, and it may be due to the absence of cross-reactivity between RHSs of different families [6].

Proteomic studies revealed that RHS proteins are expressed in epimastigotes of *T. cruzi* [68,69]. More recently, approximately 39 RHS isoforms expressed in *T. cruzi* trypomastigotes have been identified [70]. However, the diversity of RHS proteins detected by immunoblotting was more restricted, since only eight RHS isoforms were observed in this study [71]. The absence of reactivity of anti-RHS antibodies generated against the carboxy-terminal domain of RHS (TcCLB.511055.20) of CLB with *T. cruzi* trypomastigotes suggests that RHS proteins carrying the epitopes used in the mice immunization were not expressed in this developmental form. RHS proteins seem to be constitutively expressed in *T. brucei*, but they are more abundant in the procyclic forms of this parasite [6]. More recently, it has been reported that several RHSs are stage-specific regulated [10].

Since RHS is a target for the insertion of retrotransposons, the participation of RHS in controlling the expansion of these mobile elements has been proposed. Other functions for RHS have been related to *T. brucei*. TbRRM, a modulator of the chromatin structure in *T. brucei*, interacts with RHS transcripts, proteins and histones, suggesting that the RHS family could be involved in chromatin modeling [10]. Recently, it has been reported that several RHS proteins (RHS2, RHS4, and RHS6) may act as factors involved in the transcription elongation and mRNA export in *T. brucei* [11].

Little is known about the role of RHS in the *T. cruzi* life cycle. *T. cruzi* RHS proteins have been identified in the secretome of epimastigotes, trypomastigotes, and amastigotes, indicating that they are exported to the extracellular medium [71,72,73,74]. Bautista-Lopez et al., 2017 [71] showed that RHS proteins were present in the extracellular vesicles (EVs) released by *T. cruzi* trypomastigotes and amastigotes in infected Vero cells. The secreted RHS proteins reacted with sera from chronic chagasic patients ranging from asymptomatic to advanced cardiomyopathy. EVs are important modulators of the mammalian host—*T. cruzi* relationships, such as heart parasitism, susceptibility to infection of mammalian cells, and inflammatory response [72,75]. The immunoreactivity of RHSs from EVs suggests that they could participate, possibly as adjuvants, in the interaction of *T. cruzi* with the mammalian host. In this context, it is noteworthy that RHS is more abundant in the *T. cruzi* strains infective for humans (Bug2148, Y, and Sylvio X10) than in B7, which is not infective in humans [44].

In conclusion, our data suggest that unequal mitotic crossing-over and gene conversion play a significant role in shaping the patterns of homology between the RHS paralogous repeats that accelerate the generation of diversity within this multigene family. Recombination among transcribed RHS genes leads to the generation of multiple chimeric functional RHS genes. Finally, we showed the nuclear location of RHS in the replicative forms of *T. cruzi*. Although evidence for the functions of RHS in *T. cruzi* has been elusive, we suggest that these proteins could play a role in modulating the chromatin structure at the transcriptional and posttranscriptional levels, as has been suggested in *T. brucei* [10,11].

## Figures and Tables

**Table 1:** Distribution of the members of RHS groups across the chromosomes of clone CLB.

Group	Gene ID TriTrypDB ^1^	CDS (bp) ^2^	Peptide (aa) ^3^	Direction of Transcription ^4^	Subtelomeric Region ^5^	Chromosome ^6^
1	TcCLB.511845.10	270	90	Sense	-	TcChr20-P (580,762–581,031)
	TcCLB.509717.176	402	134	Sense	-	TcChr4-P (157,230–157,631)
	TcCLB.509295.90	771	256	Sense	Tel 6	TcChr28-P (746,714–747,484)
	TcCLB.510479.11	1701	567	Sense	-	TcChr38-P (1,335,682–1,337,382)
	TcCLB.506961.10	1929	642	Anti-Sense	-	TcChr18-S (118–2046)
	TcCLB.506001.90	2763	920	Sense	-	TcChr4-P (166,550–169,312)
	TcCLB.507167.70	2772	923	Sense	Tel 6	TcChr28-P (837,994–840,765)
	TcCLB.508479.500	2892	963	Anti-Sense	-	TcChr40-P (1,914,173–1,917,064)
2	TcCLB.509875.11	819	273	Sense	Tel 13	TcChr26-P (793,295–794,113)
	TcCLB.509873.10	831	276	Sense	Tel 13	TcChr26-P (794,215–795,045)
	TcCLB.508285.10	1767	588	Sense	Tel 3	TcChr19-S (653,962–655,728)
	TcCLB.506421.10	1038	345	Anti-Sense	Tel 49	TcChr31-P (53,479–54,51)
	TcCLB.509915.60	1767	588	Anti-Sense	Tel 49	TcChr31-P (64,469–66,235)
	TcCLB.506443.150	2400	799	Sense	Tel 24	TcChr11-P (510,464–512,863)
	TcCLB.507555.80	2757	918	Anti-Sense	Tel 35	TcChr35-S (510,464–512,863)
3	TcCLB.459199.10	2820	939	Anti-Sense	Tel 28	TcChr15-P (5578–8397)
	TcCLB.506047.20	1815	604	Sense	Tel 9	TcChr35-S (1,183,688–1,185,502)
	TcCLB.506017.51	1122	374	Sense	-	TcChr29-P (869,711–870,832)
	TcCLB.507167.20	2835	944	Sense	Tel 6	TcChr28-P (849,015–851,849)
	TcCLB.507611.10	2841	946	Anti-Sense	Tel 17	TcChr37-S (1391–4231)
	TcCLB.506393189	2274	758	Sense	-	TcChr14-P (596,251–598,524)
	TcCLB.506323.30	2790	929	Anti-Sense	Tel 4	TcChr22-P (62,292–65,081)
	TcCLB.509429.4	2613	871	Sense	-	TcChr6-P (364,778–367,390)
	TcCLB.511773.110	2472	995	Anti-Sense	-	TcChr17-P (301–2772)
	TcCLB.508037.10	1146	381	Anti-Sense	Tel 48	TcChr27-S (1297–2442)
	TcCLB.511929.30	2781	926	Sense	-	TcChr25-P (736,933–739,713)
	TcCLB.504109.200	3294	1097	Anti-Sense	-	TcChr39-P (599–3892)
	TcCLB.508473.10	4512	1503	Sense	Tel 30	TcChr39-S (1,847,980–1,852,491)
	TcCLB.507625.10	4149	1382	Sense	Tel 45	TcChr40-S (1,133,828–1,137,976)
	TcCLB.39997.10	1053	350	Anti-Sense	-	TcChr37-P (33,320–34,372)
4	TcCLB.504343.30	1779	592	Anti-Sense	-	TcChr7-S (60,071–61,849)
	TcCLB.507.907.30	1779	592	Anti-Sense	-	TcChr7-S (73,533–75,311)
	TcCLB.507.907.60	1779	592	Anti-Sense	-	TcChr7-S (62,859–64,637)
	TcCLB.505207.30	1626	541	Anti-Sense	-	TcChr41-P (8244–9869)
5	TcCLB.511019.80 *	1500	499	Sense	-	TcChr35-P (101,616–103,187)
	TcCLB.503881.30	1509	502	Sense	-	TcChr33-S (730,729–732,237)
	TcCLB.508119.140	1503	500	Anti-Sense	-	TcChr33-P (724,554–726,056)
	TcCLB.511907.330	1503	500	Sense	-	TcChr26-P (250,686–252,188)
	TcCLB.506529.680	444	148	Sense	-	TcChr6-S (201,683–202,126
	TcCLB.510889.352	510	170	Sense	-	TcChr6-P (201,577–202,086
6	TcCLB.509085.120	1896	631	Anti-Sense	-	TcChr15-P (164,566–166,461
	TcCLB.509437.110	1896	631	Sense	-	TcChr15-P (256,827–258,722
	TcCLB.509349.20	1893	630	Anti-Sense	Tel 2	TcChr11-S (115,973–117,865)
	TcCLB.508479.80	1947	648	Sense	-	TcChr40-P (1,993,454–1,995,400)
	TcCLB.509163.110	1962	653	Sense	-	TcChr35-P (1,138,639–1,140,600)
	TcCLB.511871.130	1896	631	Sense	-	TcChr15-S (101,636–103,531)
	TcCLB.511861.90	1896	631	Sense	-	TcChr15-P (118,605–120,500)
	TcCLB.511863.4	1572	524	Sense	-	TcChr15-P (101,616–103,187)
7	TcCLB.506809.5	354	117	Sense	-	TcChr16-P (453,466–453,819)
	TcCLB.509575.10	2763	920	Sense	-	TcChr16-P (389,424–392,186)
	TcCLB.424771.10	873	290	Sense	-	TcChr16-P (477,440–478,312)
	TcCLB.507843.10	1779	592	Sense	-	TcChr16-S (390,540–392,318)
	TcCLB.509827.4	962	320	Sense	-	TcChr16-S (389,477–390,438)
	TcCLB.507841.14	2562	854	Sense	-	TcChr16-S (452,820–455,381)
8	TcCLB.511019.13	1548	516	Sense	-	TcChr35-P (446,350–447,897)
	TcCLB.509219.20	3633	1210	Sense	-	TcChr20-P (567,813–571,445)
	TcCLB.506271.30	324	108	Sense	-	TcChr20-P (586,959–587,282)
	TcCLB.510643.190	2496	831	Sense	-	TcChr16-P (642,806–645,301)
	TcCLB.505997.60	2316	771	Anti-Sense	Tel 1	TcChr9-P (12,280–14,595)
	TcCLB.506595.149	2465	821	Anti-Sense	-	TcChr33-P (101–2565)
	TcCLB.511371.10	1785	594	Sense	-	TcChr5-S (200,095–201,879)
	TcCLB.511415.11	1095	365	Anti-Sense	-	TcChr9-S (30,121–31,215)
	TcCLB.508559.90	1821	606	Sense	Tel 21	TcChr25-S (700,188–702,008)
	TcCLB.511585.320	1932	643	Anti-Sense	Tel 14	TcChr33-S (31,331–33,262)
	TcCLB.507015.10 *	2988	995	Anti-Sense	Tel 10	TcChr13-P (1626–4613)
	TcCLB.509917.19	1815	605	Anti-Sense	Tel 49	TcChr31-P (54,619–56,433)
9	TcCLB.503401.11	243	81	Sense	-	TcChr22-S (214,572–214,814)
	TcCLB.506629.240	327	109	Anti-Sense	-	TcChr39-P (389,442–389,768)
	TcCLB.509829.9	909	303	Anti-Sense	-	TcChr39-S 392,244–393)
	TcCLB.509329.9	752	250	Sense	-	TcChr22-P (339,264–340,015)
	TcCLB.509463.41 *	1209	403	Anti-Sense	-	TcChr22-P (391,811–393,019)
	TcCLB.509843.10	1503	500	Sense	-	TcChr22-S (214,918–216,420)
10	TcCLB.506139.200	1674	557	Sense	-	TcChr18-P (357,746–359,419)
	TcCLB.510845.10	1824	608	Anti-Sense	-	TcChr19-S (28,739–30,562)

^1^ TriTrypDB [41]. ^2^ CDS (coding DNA sequence), size in bp. ^3^ The translated peptide, size in amino acid (aa). ^4^ The direction of transcription. ^5^ RHS is located in the subtelomeric regions of the chromosomes of clone CLB [45]. ^6^ Genomic coordinates at the in silico chromosome of clone CLB (TcChr) [40]. * The other allele at the same locus is a pseudogene.

## Supplementary Materials

The following are available online at https://www.mdpi.com/2073-4425/11/9/1085/s1, Figure S1: Flowchart of RHS sequences’ identification, and quality validation; Figure S2: Integrity and purity of RHS recombinant protein; Figure S3: Distribution of RHS sequences across the chromosomes of clone CLB of *T. cruzi*; Figure S4: Proportion of total RHS length in each chromosome of clone CLB; Figure S5: Phylogeny and classification of transcribed RHS sequences of clone CLB; Table S1. Mapping of RHS sequences on the chromosomes of clone CLB of *Trypanosoma cruzi*.

## References

[1] WHO (2020). Chagas Disease (American Trypanosomiasis). https://www.who.int/health-topics/chagas-disease.

[2] El-Sayed N.M., Myler P.J., Bartholomeu D.C., Nilsson D., Aggarwal G., Tran A.N., Ghedin E., Worthey E.A., Delcher A.L., Blandin G. (2005). The Genome Sequence of *Trypanosoma cruzi*, Etiologic Agent of Chagas Disease. Science.

[3] Berná L., Rodriguez M., Chiribao M.L., Parodi-Talice A., Pita S., Rijo G., Alvarez-Valin F., Robello C. (2018). Expanding an expanded genome: Long-read sequencing of *Trypanosoma cruzi*. Microb. Genomics.

[4] Pita S., Díaz-Viraqué F., Iraola G., Robello C. (2019). The Tritryps Comparative Repeatome: Insights on Repetitive Element Evolution in Trypanosomatid Pathogens. Genome Biol. Evol..

[5] Chiurillo M.A., Barros R.R.M., Souza R.T., Marini M.M., Antonio C.R., Cortez D.R., Curto M., Lorenzi H.A., Schijman A.G., Ramirez J.L. (2016). Subtelomeric I-SceI-mediated double-strand breaks are repaired by homologous recombination in *Trypanosoma cruzi*. Front. Microbiol..

[6] Bringaud F., Biteau N., Melville S.E., Hez S., El-Sayed N.M., Leech V., Berriman M., Hall N., Donelson J.E., Baltz T. (2002). A New, Expressed Multigene Family Containing a Hot Spot for Insertion of Retroelements Is Associated with Polymorphic Subtelomeric Regions of Trypanosoma brucei. Eukaryot. Cell.

[7] Bringaud F., Bartholomeu D.C., Blandin G., Delcher A., Baltz T., El-Sayed N.M.A., Ghedin E. (2006). The *Trypanosoma cruzi* L1Tc and NARTc Non-LTR Retrotransposons Show Relative Site Specificity for Insertion. Mol. Biol. Evol..

[8] Durand-Dubief M., Absalon S., Menzer L., Ngwabyt S., Ersfeld K., Bastin P. (2007). The Argonaute protein TbAGO1 contributes to large and mini-chromosome segregation and is required for control of RIME retroposons and RHS pseudogene-associated transcripts. Mol. Biochem. Parasitol..

[9] Wen Y.-Z., Zheng L.-L., Liao J.-Y., Wang M.-H., Wei Y., Guo X.-M., Qu L.-H., Ayala F.J., Lun Z.-R. (2011). Pseudogene-derived small interference RNAs regulate gene expression in African Trypanosoma brucei. Proc. Natl. Acad. Sci. USA.

[10] Naguleswaran A., Gunasekera K., Schimanski B., Heller M., Hemphill A., Ochsenreiter T., Roditi I. (2015). Trypanosoma brucei RRM1 Is a Nuclear RNA-Binding Protein and Modulator of Chromatin Structure. MBio.

[11] Florini F., Naguleswaran A., Gharib W.H., Bringaud F., Roditi I. (2019). Unexpected diversity in eukaryotic transcription revealed by the retrotransposon hotspot family of Trypanosoma brucei. Nucleic Acids Res..

[12] Zingales B., Pereira M.E.S., Almeida K.A., Umezawa E.S., Nehme N.S., Oliveira R.P., Macedo A., Souto R.P. (1997). Biological Parameters and Molecular Markers of Clone CL Brener—The Reference Organism of the *Trypanosoma cruzi* Genome Project. Mem. Inst. Oswaldo Cruz.

[13] Yoshida N. (1983). Surface antigens of metacyclic trypomastigotes of *Trypanosoma cruzi*. Infect. Immun..

[14] Steindel M., Pinto J.C.C., Toma H.K., Mangia R.H.R., Ribeiro-Rodrigues R., Romanha A.J. (1991). Trypanosoma rangeli (Tejera, 1920) isolated from a sylvatic rodent (*Echimys dasythrix*) in Santa Catarina island, Santa Catarina state: First report of this trypanosome in southern Brazil. Mem. Inst. Oswaldo Cruz.

[15] Camargo E.P. (1964). Growth and differentiation in *Trypanosoma cruzi*. I. Origin of metacyclic trypanosomes in liquid media. Rev. Inst. Med. Trop. Sao Paulo.

[16] Vieira da Silva C., Luquetti A.O., Rassi A., Mortara R.A. (2006). Involvement of Ssp-4-related carbohydrate epitopes in mammalian cell invasion by *Trypanosoma cruzi* amastigotes. Microbes Infect..

[17] Altschul S.F., Gish W., Miller W., Myers E.W., Lipman D.J. (1990). Basic local alignment search tool. J. Mol. Biol..

[18] Conserved Domains and Protein Classification. https://www.ncbi.nlm.nih.gov/cdd.

[19] Edgar R.C. (2004). MUSCLE: Multiple sequence alignment with high accuracy and high throughput. Nucleic Acids Res..

[20] Castresana J. (2000). Selection of Conserved Blocks from Multiple Alignments for Their Use in Phylogenetic Analysis. Mol. Biol. Evol..

[21] TriTrypDB-31_TcruziCLBrener_AnnotatedTranscripts.fast. https://tritrypdb.org/common/downloads/release-31/TcruziCLBrener/fasta/data/.

[22] Stamatakis A. (2014). RAxML version 8: A tool for phylogenetic analysis and post-analysis of large phylogenies. Bioinformatics.

[23] FigTree GitHub Repository. http://tree.bio.ed.ac.uk/software/figtree/.

[24] Martins N.O., de Souza R.T., Cordero E.M., Maldonado D.C., Cortez C., Marini M.M., Ferreira E.R., Bayer-Santos E., de Almeida I.C., Yoshida N. (2015). Molecular Characterization of a Novel Family of *Trypanosoma cruzi* Surface Membrane Proteins (TcSMP) Involved in Mammalian Host Cell Invasion. PLoS Negl. Trop. Dis..

[25] Tibayrenc M., Ward P., Moya A., Ayala F.J. (1986). Natural populations of *Trypanosoma cruzi*, the agent of Chagas disease, have a complex multiclonal structure. Proc. Natl. Acad. Sci. USA..

[26] Oliveira R.P., Broude N.E., Macedo A.M., Cantor C.R., Smith C.L., Pena S.D.J. (1998). Probing the genetic population structure of *Trypanosoma cruzi* with polymorphic microsatellites. Proc. Natl. Acad. Sci. USA.

[27] Tibayrenc M., Ayala F.J. (2002). The clonal theory of parasitic protozoa: 12 years on. Trends Parasitol..

[28] Pena S.D.J., Machado C.R., Macedo A.M. (2009). *Trypanosoma cruzi*: Ancestral genomes and population structure. Mem. Inst. Oswaldo Cruz.

[29] Gaunt M.W., Yeo M., Frame I.A., Stothard J.R., Carrasco H.J., Taylor M.C., Mena S.S., Veazey P., Miles G.A.J., Acosta N. (2003). Mechanism of genetic exchange in American trypanosomes. Nature.

[30] Machado C.A., Ayala F.J. (2001). Nucleotide sequences provide evidence of genetic exchange among distantly related lineages of *Trypanosoma cruzi*. Proc. Natl. Acad. Sci. USA.

[31] Augusto-Pinto L., Teixeira S.M.R., Pena S.D.J., Machado C.R. (2003). Single-nucleotide polymorphisms of the *Trypanosoma cruzi* MSH2 gene support the existence of three phylogenetic lineages presenting differences in mismatch-repair efficiency. Genetics.

[32] Brisse S., Henriksson J., Barnabé C., Douzery E.J.P., Berkvens D., Serrano M., De Carvalho M.R.C., Buck G.A., Dujardin J.-C., Tibayrenc M. (2003). Evidence for genetic exchange and hybridization in *Trypanosoma cruzi* based on nucleotide sequences and molecular karyotype. Infect. Genet. Evol..

[33] Westenberger S.J., Barnabé C., Campbell D.A., Sturm N.R. (2005). Two Hybridization Events Define the Population Structure of *Trypanosoma cruzi*. Genetics.

[34] de Freitas J.M., Augusto-Pinto L., Pimenta J.R., Bastos-Rodrigues L., Gonçalves V.F., Teixeira S.M.R., Chiari E., Junqueira Â.C.V., Fernandes O., Macedo A.M. (2006). Ancestral Genomes, Sex, and the Population Structure of *Trypanosoma cruzi*. PLoS Pathog..

[35] Berry A.S.F., Salazar-Sánchez R., Castillo-Neyra R., Borrini-Mayorí K., Chipana-Ramos C., Vargas-Maquera M., Ancca-Juarez J., Náquira-Velarde C., Levy M.Z., Brisson D. (2019). Sexual reproduction in a natural *Trypanosoma cruzi* population. PLoS Negl. Trop. Dis..

[36] Schwabl P., Imamura H., Van den Broeck F., Costales J.A., Maiguashca-Sánchez J., Miles M.A., Andersson B., Grijalva M.J., Llewellyn M.S. (2019). Meiotic sex in Chagas disease parasite *Trypanosoma cruzi*. Nat. Commun..

[37] Tibayrenc M. (2003). Genetic subdivisions within *Trypanosoma cruzi* (Discrete Typing Units) and their relevance for molecular epidemiology and experimental evolution. Kinetoplastid Biol. Dis..

[38] Zingales B., Andrade S., Briones M., Campbell D., Chiari E., Fernandes O., Guhl F., Lages-Silva E., Macedo A., Machado C. (2009). A new consensus for *Trypanosoma cruzi* intraspecific nomenclature: Second revision meeting recommends TcI to TcVI. Mem. Inst. Oswaldo Cruz.

[39] Zingales B., Miles M.A., Campbell D.A., Tibayrenc M., Macedo A.M., Teixeira M.M.G., Schijman A.G., Llewellyn M.S., Lages-Silva E., Machado C.R. (2012). The revised *Trypanosoma cruzi* subspecific nomenclature: Rationale, epidemiological relevance and research applications. Infect. Genet. Evol..

[40] Weatherly D.B., Boehlke C., Tarleton R.L. (2009). Chromosome level assembly of the hybrid *Trypanosoma cruzi* genome. BMC Genom..

[41] TriTrypDB The Kinetoplastid Genomics Resource. https://tritrypdb.org/tritrypdb/.

[42] El-Sayed N.M., Myler P.J., Blandin G., Berriman M., Crabtree J., Aggarwal G., Caler E., Renauld H., Worthey E.A., Hertz-Fowler C. (2005). Comparative genomics of trypanosomatid parasitic protozoa. Science.

[43] Azuaje F.J., Ramirez J.L., Da Silveira J.F. (2007). In silico, biologically-inspired modelling of genomic variation generation in surface proteins of *Trypanosoma cruzi*. Kinetoplastid Biol. Dis..

[44] Callejas-Hernández F., Rastrojo A., Poveda C., Gironès N., Fresno M. (2018). Genomic assemblies of newly sequenced *Trypanosoma cruzi* strains reveal new genomic expansion and greater complexity. Sci. Rep..

[45] Moraes Barros R.R., Marini M.M., Antônio C., Cortez D.R., Miyake A.M., Lima F.M., Ruiz J.C., Bartholomeu D.C., Chiurillo M.A., Ramirez J. (2012). Anatomy and evolution of telomeric and subtelomeric regions in the human protozoan parasite *Trypanosoma cruzi*. BMC Genomics.

[46] Chiurillo M.A., Regina Antonio C., Mendes Marini M., de Souza R.T., Franco da Silveira J. (2017). Chromosomes Ends and Telomere Biology of Trypanosomatids. Frontiers in Parasitology.

[47] Ghedin E., Bringaud F., Peterson J., Myler P., Berriman M., Ivens A., Andersson B., Bontempi E., Eisen J., Angiuoli S. (2004). Gene synteny and evolution of genome architecture in trypanosomatids. Mol. Biochem. Parasitol..

[48] Kim D., Chiurillo M.A., El-Sayed N., Jones K., Santos M.R.M., Porcile P.E., Andersson B., Myler P., da Silveira J.F., Ramírez J.L. (2005). Telomere and subtelomere of *Trypanosoma cruzi* chromosomes are enriched in (pseudo)genes of retrotransposon hot spot and trans-sialidase-like gene families: The origins of *T. cruzi* telomeres. Gene.

[49] Bartholomeu D.C., Cerqueira G.C., Leão A.C.A., DaRocha W.D., Pais F.S., Macedo C., Djikeng A., Teixeira S.M.R., El-Sayed N.M. (2009). Genomic organization and expression profile of the mucin-associated surface protein (masp) family of the human pathogen *Trypanosoma cruzi*. Nucleic Acids Res..

[50] Martins C., Baptista C.S., Ienne S., Cerqueira G.C., Bartholomeu D.C., Zingales B. (2008). Genomic organization and transcription analysis of the 195-bp satellite DNA in *Trypanosoma cruzi*. Mol. Biochem. Parasitol..

[51] Ramirez J.L. (2020). An Evolutionary View of *Trypanosoma cruzi* Telomeres. Front. Cell. Infect. Microbiol..

[52] Zolan M.E. (1995). Chromosome-length polymorphism in fungi. Microbiol. Rev..

[53] Symington L.S., Rothstein R., Lisby M. (2014). Mechanisms and Regulation of Mitotic Recombination in Saccharomyces cerevisiae. Genetics.

[54] dos Santos Júnior A.D.C.M., Kalume D.E., Camargo R., Gómez-Mendoza D.P., Correa J.R., Charneau S., de Sousa M.V., de Lima B.D., Ricart C.A.O. (2015). Unveiling the *Trypanosoma cruzi* Nuclear Proteome. PLoS ONE.

[55] Thon G., Baltz T., Eisen H. (1989). Antigenic diversity by the recombination of pseudogenes. Genes Dev..

[56] Barry J.D., Ginger M.L., Burton P., McCulloch R. (2003). Why are parasite contingency genes often associated with telomeres?. Int. J. Parasitol..

[57] Marcello L., Barry J.D. (2007). Analysis of the VSG gene silent archive in Trypanosoma brucei reveals that mosaic gene expression is prominent in antigenic variation and is favored by archive substructure. Genome Res..

[58] Hall J.P.J., Wang H., Barry J.D. (2013). Mosaic VSGs and the Scale of Trypanosoma brucei Antigenic Variation. PLoS Pathog..

[59] Roth C., Bringaud F., Layden R.E., Baltz T., Eisen H. (1989). Active late-appearing variable surface antigen genes in Trypanosoma equiperdum are constructed entirely from pseudogenes. Proc. Natl. Acad. Sci. USA.

[60] Callejas S., Leech V., Reitter C., Melville S. (2006). Hemizygous subtelomeres of an African trypanosome chromosome may account for over 75% of chromosome length. Genome Res..

[61] Takle G.B., O’Connor J., Young A.J., Cross G.A.M. (1992). Sequence homology and absence of mRNA defines a possible pseudogene member of the *Trypanosoma cruzi* gp85/sialidase multigene family. Mol. Biochem. Parasitol..

[62] Taylor M.C., Muhia D.K., Baker D.A., Mondragon A., Schaap P., Kelly J.M. (1999). *Trypanosoma cruzi* adenylyl cyclase is encoded by a complex multigene family. Mol. Biochem. Parasitol..

[63] Allen C.L., Kelly J.M. (2001). *Trypanosoma cruzi*: Mucin Pseudogenes Organized in a Tandem Array. Exp. Parasitol..

[64] Cerqueira G.C., Bartholomeu D.C., DaRocha W.D., Hou L., Freitas-Silva D.M., Machado C.R., El-Sayed N.M., Teixeira S.M.R. (2008). Sequence diversity and evolution of multigene families in *Trypanosoma cruzi*. Mol. Biochem. Parasitol..

[65] Jackson A.P. (2007). Tandem gene arrays in Trypanosoma brucei: Comparative phylogenomic analysis of duplicate sequence variation. BMC Evol. Biol..

[66] de Araujo C.B., da Cunha J.P.C., Inada D.T., Damasceno J., Lima A.R.J., Hiraiwa P., Marques C., Gonçalves E., Nishiyama-Junior M.Y., McCulloch R. (2020). Replication origin location might contribute to genetic variability in *Trypanosoma cruzi*. BMC Genomics.

[67] Weir W., Capewell P., Foth B., Clucas C., Pountain A., Steketee P., Veitch N., Koffi M., de Meeûs T., Kaboré J. (2016). Population genomics reveals the origin and asexual evolution of human infective trypanosomes. Elife.

[68] Parodi-Talice A., Durán R., Arrambide N., Prieto V., Piñeyro M.D., Pritsch O., Cayota A., Cerveñansky C., Robello C. (2004). Proteome analysis of the causative agent of Chagas disease: *Trypanosoma cruzi*. Int. J. Parasitol..

[69] Parodi-Talice A., Monteiro-Goes V., Arrambide N., Avila A.R., Duran R., Correa A., Dallagiovanna B., Cayota A., Krieger M., Goldenberg S. (2007). Proteomic analysis of metacyclic trypomastigotes undergoing *Trypanosoma cruzi* metacyclogenesis. J. Mass Spectrom..

[70] Brunoro G.V.F., Caminha M.A., da Silva Ferreira A.T., da Veiga Leprevost F., Carvalho P.C., Perales J., Valente R.H., Menna-Barreto R.F.S. (2015). Reevaluating the *Trypanosoma cruzi* proteomic map: The shotgun description of bloodstream trypomastigotes. J. Proteomics.

[71] Bautista-López N.L., Ndao M., Camargo F.V., Nara T., Annoura T., Hardie D.B., Borchers C.H., Jardim A. (2017). Characterization and Diagnostic Application of *Trypanosoma cruzi* Trypomastigote Excreted-Secreted Antigens Shed in Extracellular Vesicles Released from Infected Mammalian Cells. J. Clin. Microbiol..

[72] Trocoli Torrecilhas A.C., Tonelli R.R., Pavanelli W.R., da Silva J.S., Schumacher R.I., de Souza W., e Silva N.C., de Almeida Abrahamsohn I., Colli W., Manso Alves M.J. (2009). *Trypanosoma cruzi*: Parasite shed vesicles increase heart parasitism and generate an intense inflammatory response. Microbes Infect..

[73] Bayer-Santos E., Aguilar-Bonavides C., Rodrigues S.P., Cordero E.M., Marques A.F., Varela-Ramirez A., Choi H., Yoshida N., Da Silveira J.F., Almeida I.C. (2013). Proteomic analysis of *Trypanosoma cruzi* secretome: Characterization of two populations of extracellular vesicles and soluble proteins. J. Proteome Res..

[74] Brossas J.Y., Gulin J.E.N., Bisio M.M.C., Chapelle M., Marinach-Patrice C., Bordessoules M., Ruiz G.P., Vion J., Paris L., Altcheh J. (2017). Secretome analysis of *Trypanosoma cruzi* by proteomics studies. PLoS ONE.

[75] Garcia-Silva M.R., Cura Das Neves R.F., Cabrera-Cabrera F., Sanguinetti J., Medeiros L.C., Robello C., Naya H., Fernandez-Calero T., Souto-Padron T., De Souza W. (2014). Extracellular vesicles shed by *Trypanosoma cruzi* are linked to small RNA pathways, life cycle regulation, and susceptibility to infection of mammalian cells. Parasitol. Res..

