# Supplementary material for: Genomic Organization and Generation of Genetic Variability in the RHS (Retrotransposon Hot Spot) Protein Multigene Family in Trypanosoma cruzi
Source: Genes (Basel). 2020 Sep 17;11(9):1085. doi: 10.3390/genes11091085 (PMC7563717; doi:10.3390/genes11091085)
Supplement: Supplementary file 1 [file genes-11-01085-s001.zip › Supplementary_Figures_x.pdf]

Article: Genomic organization and generation of genetic variability in the RHS (retrotransposon hot spot) protein multigene family in Trypanosoma cruzi

Werica P. Bernardo[1]; Renata T. Souza[1#]; Andre G. Costa-Martins[2#]; Eden R. Ferreira[1]; Renato A. Mortara[1]; Marta M.G. Teixeira[2]; José L. Ramirez[3*]; José F. Da Silveira[1]*.

[1] Department of Microbiology, Immunology and Parasitology, Escola Paulista de Medicina, Universidade Federal de São Paulo, São Paulo – SP, 04023-062, Brazil.

[2] Department of Parasitology, Instituto de Ciências Biomédicas, Universidade de São Paulo, São Paulo - SP, 05508-000, Brazil.

[3] Fundación Instituto de Estudios Avanzados (IDEA) and Universidad Central de Venezuela - Caracas 1080, Distrito Capital, Venezuela.

[#] Present address: Universidade Cruzeiro do Sul, São Paulo - SP, 08060-070, Brazil (RTS); Department of Clinical and Toxicological Analyses, Faculdade de Ciências Farmacêuticas, Universidade de São Paulo, São Paulo- SP, 05508-000, Brazil (A.G.C.M).
* Correspondence: jose.franco@unifesp.br (JFDS); ramjoseluis@gmail.com (JLR).

Supplementary Materials: The following are available online at www.mdpi.com/xxx/s1,

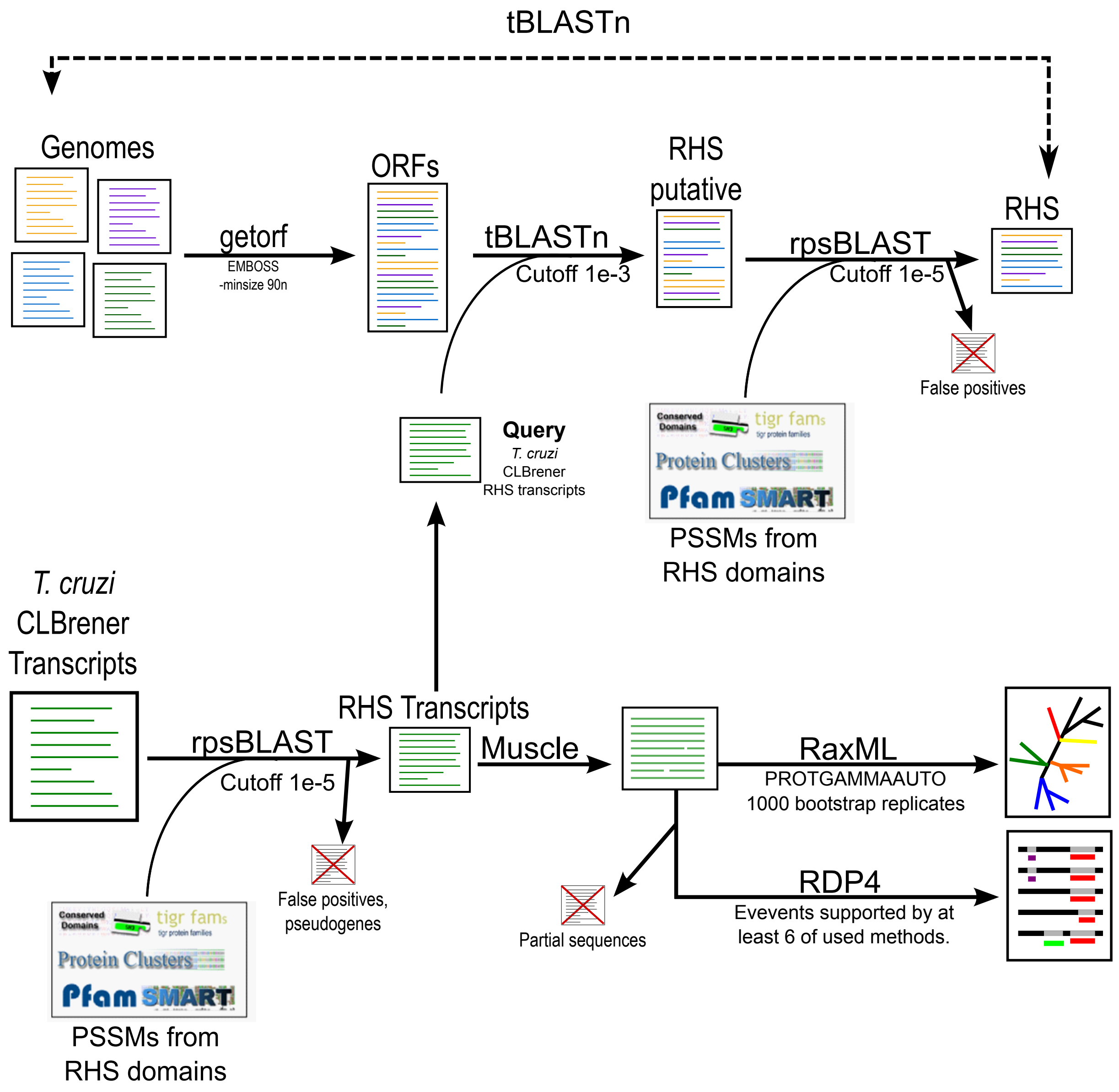

Figure S1: Flowchart of RHS sequences identification and quality validation. RHS Transcripts from T. cruzi (CLBrener strain) were obtained using rps-BLAST against NCBI Conserved Domain Database (CDD). Only transcripts presenting RHS domain architecture recovered with E-value less than 1e-5 were considered for further analysis. Sequences marked as pseudo-genes (displaying internal stop codons) were not included in phylogeny and recombination analysis, while partial sequences were also excluded from recombination analysis. Selected transcripts from T. cruzi (CLBrener strain) were used as query to drive tBLASTn searches against ORFs obtained from non annotated draft genomes. Retrieved ORFs were curated for RHS domain using rps-BLAST and CDD.

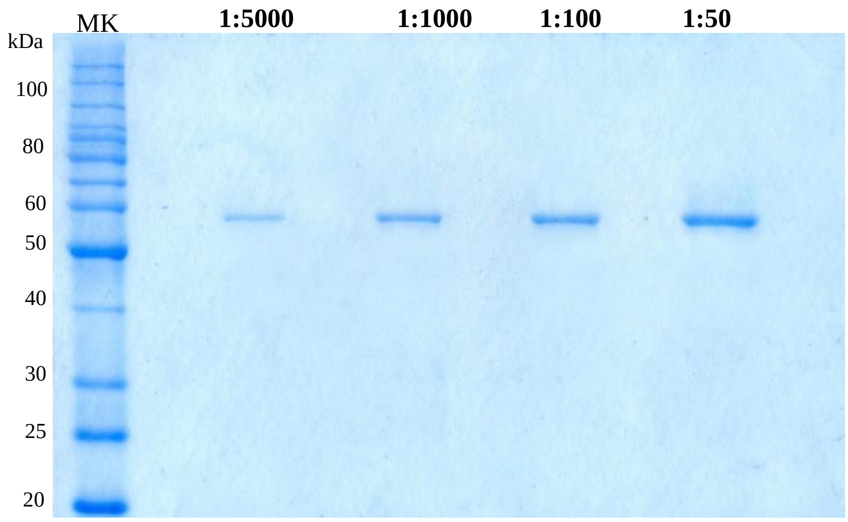

Figure S2. Integrity and purity of RHS recombinant protein. Samples containing different dilutions of the purified RHS protein were separated by SDS-PAGE (10%) and stained with Coomassie blue. MK, BenchMark Protein Ladder.

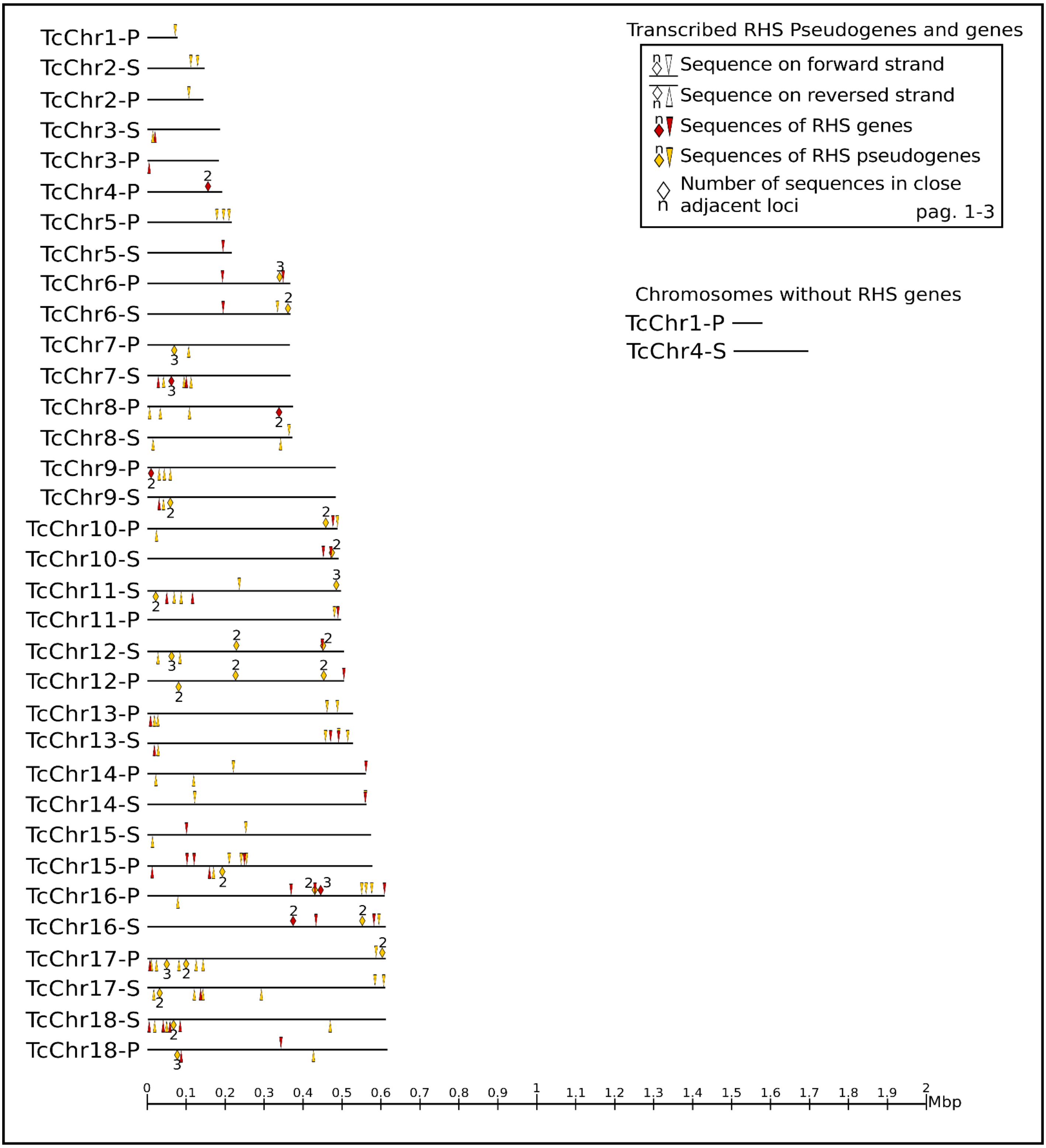

Transcribed RHS Pseudogenes and genes
Sequence on forward strand
Sequence on reversed strand
Sequences of RHS genes
Sequences of RHS pseudogenes
Number of sequences in close adjacent loci
pag. 1-3
Chromosomes without RHS genes
TcChr1-P
TcChr4-S
TcChr1-P
TcChr2-S
TcChr2-P
TcChr3-S
TcChr3-P
TcChr4-P
TcChr5-P
TcChr5-S
TcChr6-P
TcChr6-S
TcChr7-P
TcChr7-S
TcChr8-P
TcChr8-S
TcChr9-P
TcChr9-S
TcChr10-P
TcChr10-S
TcChr11-S
TcChr11-P
TcChr12-S
TcChr12-P
TcChr13-P
TcChr13-S
TcChr14-P
TcChr14-S
TcChr15-S
TcChr15-P
TcChr16-P
TcChr16-S
TcChr17-P
TcChr17-S
TcChr18-S
TcChr18-P
0 0.1 0.2 0.3 0.4 0.5 0.6 0.7 0.8 0.9 1 1.1 1.2 1.3 1.4 1.5 1.6 1.7 1.8 1.9 2
Mbp

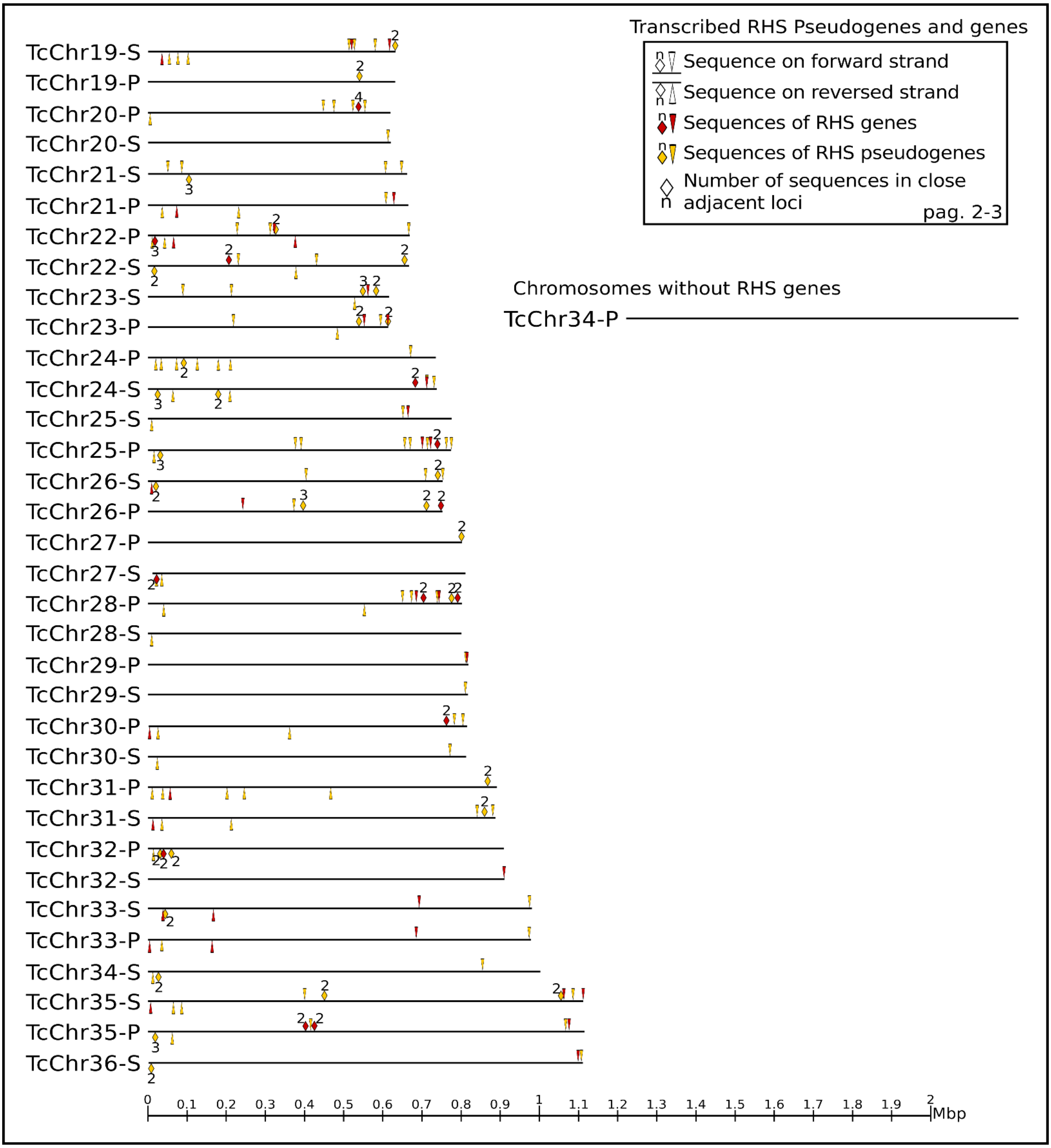
Transcribed RHS Pseudogenes and genes
Sequence on forward strand
Sequence on reversed strand
Sequences of RHS genes
Sequences of RHS pseudogenes
Number of sequences in close adjacent loci
pag. 2-3
Chromosomes without RHS genes
TcChr34-P
TcChr19-S
TcChr19-P
TcChr20-P
TcChr20-S
TcChr21-S
TcChr21-P
TcChr22-P
TcChr22-S
TcChr23-S
TcChr23-P
TcChr24-P
TcChr24-S
TcChr25-S
TcChr25-P
TcChr26-S
TcChr26-P
TcChr27-P
TcChr27-S
TcChr28-P
TcChr28-S
TcChr29-P
TcChr29-S
TcChr30-P
TcChr30-S
TcChr31-P
TcChr31-S
TcChr32-P
TcChr32-S
TcChr33-S
TcChr33-P
TcChr34-S
TcChr35-S
TcChr35-P
TcChr36-S
0
0.1
0.2
0.3
0.4
0.5
0.6
0.7
0.8
0.9
1
1.1
1.2
1.3
1.4
1.5
1.6
1.7
1.8
1.9
2
Mbp

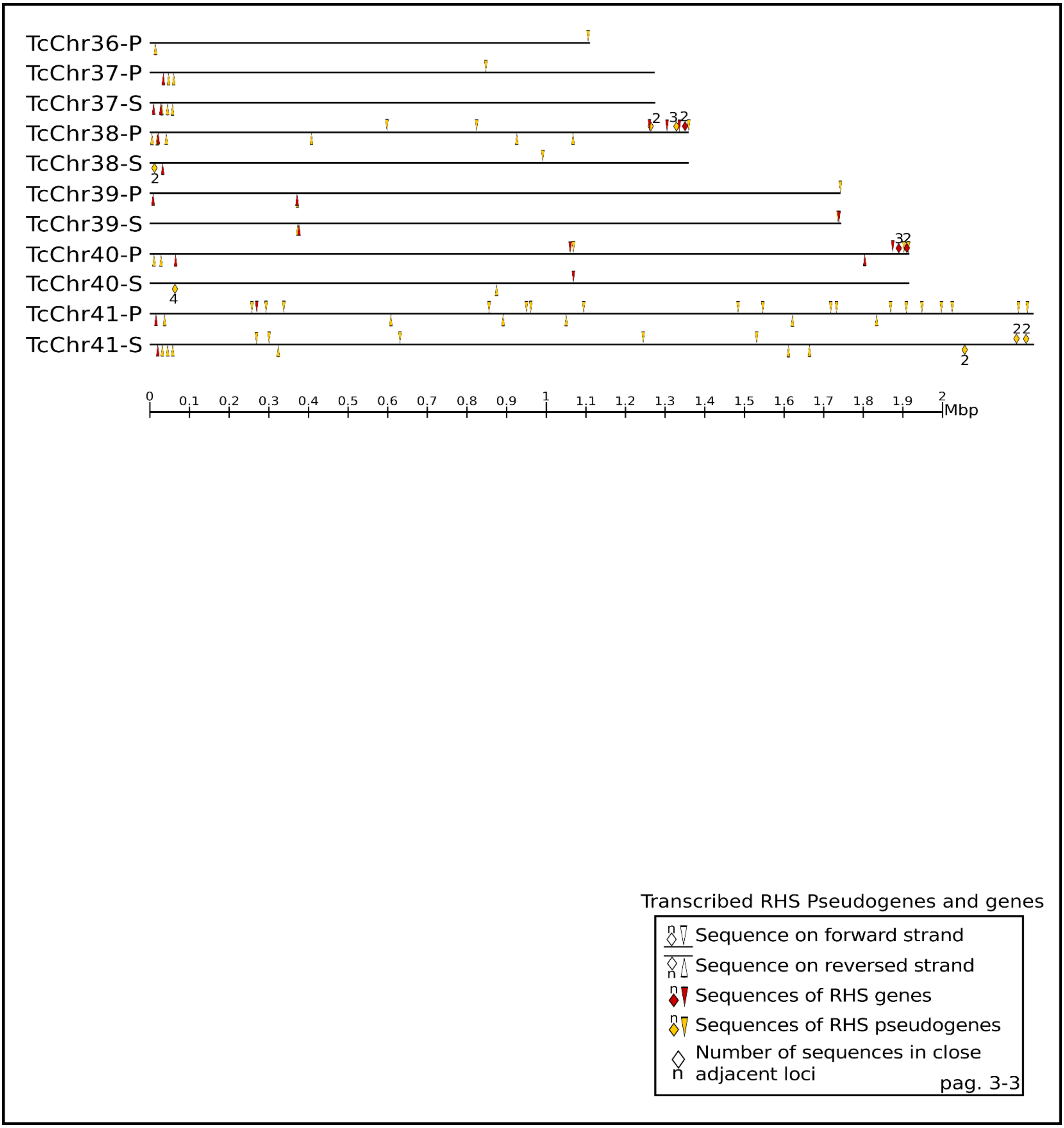

Figure S3: Distribution of RHS sequences across the chromosomes of clone CLB of *T. cruzi*. RHS sequences were mapped on both haplotypes (S, Esmeraldo like and P, Non-Esmeraldo like) on each *in silico* chromosome of CLB reference genome (Weatherly et al., 2009). The chromosomes of CLB are numbered **from 1 to 41,** from the smallest TcChr1 (0.77 Mb) to the largest TcChr41 (2.37 Mb), and they are represented to scale.

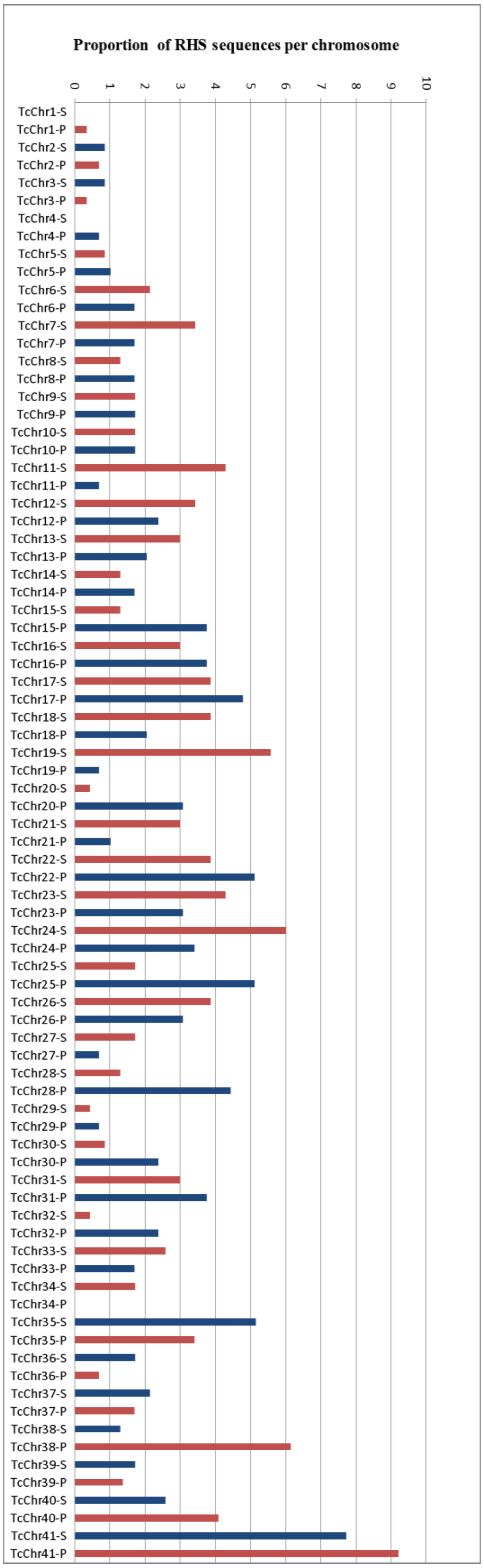

Figure S4: Proportion of total RHS length in each chromosome of clone CLB. The proportion was estimated summing the length (bp) of RHS genes and pseudogenes and dividing by the chromosome size. The haplotypes S and P are denoted by blue and red, respectively. Proportion is on the Y axis.

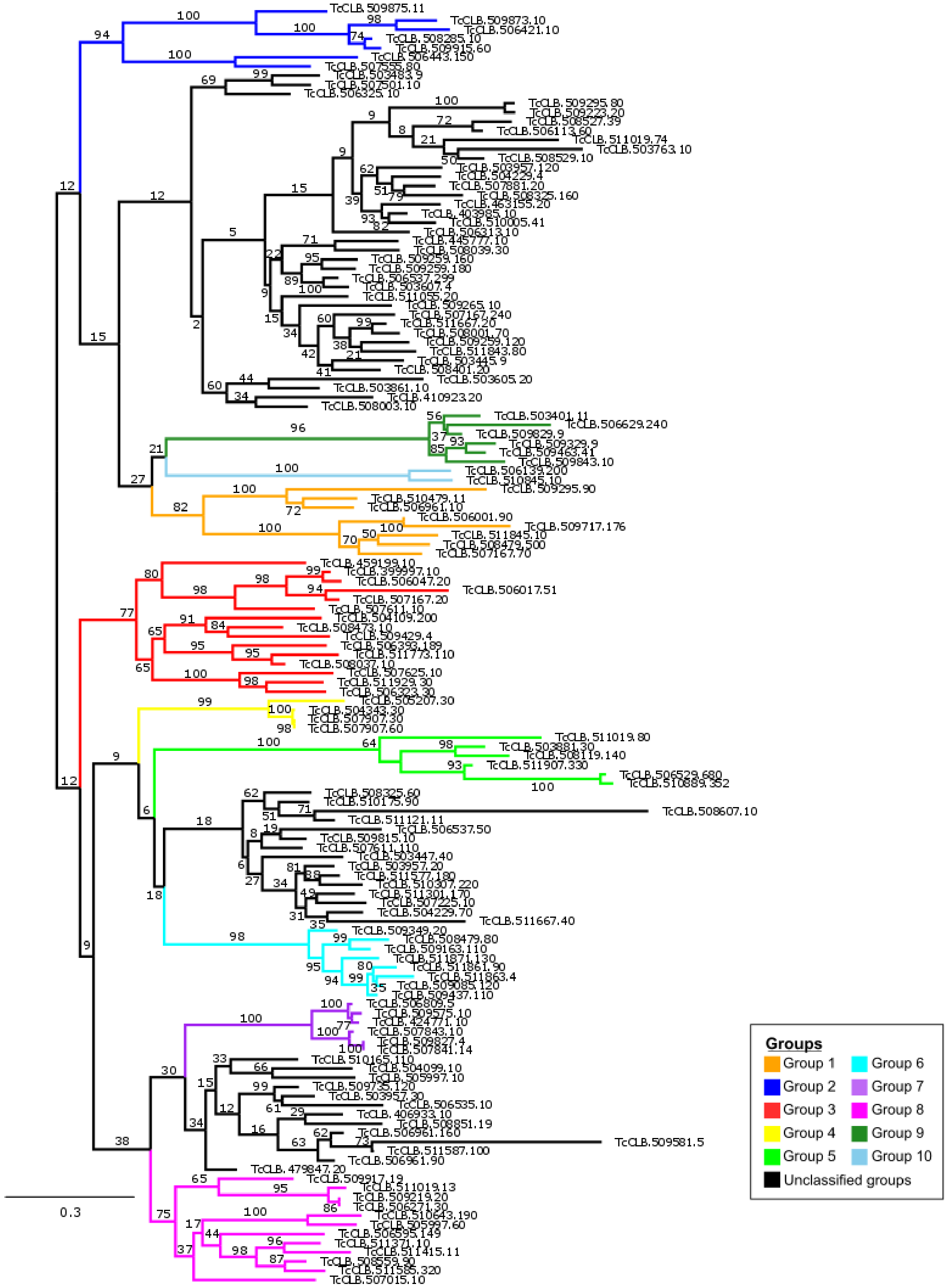

Figure S5: Phylogeny and classification of transcribed RHS sequences of clone CLB. The results are shown in the format rooted in the midpoint. Sequences are indicated by their access number in the TriTrypDB.
